# Depletion of the *N*^*6*^-Methyladenosine (m6A) reader protein IGF2BP3 induces ferroptosis in glioma by modulating the expression of GPX4

**DOI:** 10.1038/s41419-024-06486-z

**Published:** 2024-03-01

**Authors:** Limei Deng, Yunbo Di, Caiyun Chen, Juan Xia, Bingxi Lei, Ning Li, Qingyu Zhang

**Affiliations:** 1 Laboratory of Obstetrics and Gynecology, Affiliated Hospital of Guangdong Medical University, Zhanjiang, Guangdong 524001 China; 2 The Marine Biomedical Research Institute, Guangdong Medical University, Zhanjiang, 524023 China; 3 Department of Hematology, Affiliated Hospital of Guangdong Medical University, Zhanjiang, Guangdong 524001 China; 4 Department of Neurosurgery, Sun Yat-Sen Memorial Hospital, Sun Yat-Sen University, Guangzhou, 510120 China

**Keywords:** CNS cancer, Methylation

## Abstract

Emerging evidence highlights the multifaceted contributions of m6A modifications to glioma. IGF2BP3, a m6A modification reader protein, plays a crucial role in post-transcriptional gene regulation. Though several studies have identified IGF2BP3 as a poor prognostic marker in glioma, the underlying mechanism remains unclear. In this study, we demonstrated that IGF2BP3 knockdown is detrimental to cell growth and survival in glioma cells. Notably, we discovered that IGF2BP3 regulated ferroptosis by modulating the protein expression level of GPX4 through direct binding to a specific motif on GPX4 mRNA. Strikingly, the m6A modification at this motif was found to be critical for GPX4 mRNA stability and translation. Furthermore, IGF2BP3 knockdown glioma cells were incapable of forming tumors in a mouse xenograft model and were more susceptible to phagocytosis by microglia. Our findings shed light on an unrecognized regulatory function of IGF2BP3 in ferroptosis. The identification of a critical m6A site within the GPX4 transcript elucidates the significance of post-transcriptional control in ferroptosis.

## Introduction

Glioma is a type of tumor that originates in the glial cells of the brain or spinal cord. Traditionally, grading of central nervous system (CNS) tumors has been reliant on histological characteristics. Within this context, the most prevalent malignant primary brain tumor is glioblastoma (GBM), classified by the World Health Organization (WHO) as a grade IV glioma [1]. Cases of WHO grade II and grade III tumors are classified as lower grade gliomas (LGG) [1]. However, modern classification incorporates molecular markers like IDH, 1p/19q, TERT, and TP53 status, enabling enhanced diagnostic precision and prognostic accuracy [2–8]. These classifications also help in formulating personalized therapeutic approaches for patients with specific molecular characteristics. Hence, further research into the molecular genetic features of glioma is anticipated to improve diagnostic accuracy, prognostic predication, and the customization of therapeutic strategies for individuals with glioma.

*N*^6^-Methyladenosine (m6A) is the most common type of RNA modification that occurs through methylation at the N6-position of adenosine nucleotides. This modification exerts profound effects on RNA splicing, translation, stability, RNA-protein interactions, and subcellular localization [9–14]. The m6A modification in mRNA is dynamically modulated by a cohort of enzymes: The methyl group is transferred onto adenosine in RNA by methyltransferases (writers), removed by demethylases (erasers), and recognized by m6A-binding proteins (readers) [15]. Growing evidence demonstrates the pivotal role of m6A modifications in mRNA across various cancers, with emerging research indicating m6A regulators as potential diagnostic and therapeutic targets in cancers [16–20]. The m6A reader proteins specifically recognize and bind to m6A-modified RNA molecules, thereby translating the chemical modification into functional outcomes. Studies on m6A readers help to elucidate the mechanism of how m6A modification impact gene expression, cellular responses, and disease processes.

Emerging research has revealed that m6A modifications play a multifaceted role in glioma biology, influencing glioblastoma stem cells (GSCs) maintenance, tumor progression, therapy resistance, and prognostic outcomes [21–27]. Understanding the comprehensive interplay between m6A regulators in glioma has the potential to uncover novel diagnostic and therapeutic targets, ultimately leading to enhanced outcomes for patients. However, it’s important to note that this field is still evolving, and further research is needed to fully elucidate the precise mechanisms and therapeutic implications of m6A modification in glioma.

Ferroptosis is a form of regulated cell death characterized by iron-dependent accumulation of lipid peroxides, leading to oxidative stress-induced cell death [28]. Glutathione peroxidase 4 (GPX4) is a critical enzyme that protects cells from ferroptosis by reducing lipid hydroperoxides [29]. Ferroptosis has garnered substantial attention as a promising therapeutic target, particularly given the heightened demand for iron by cancer cells to sustain their rapid proliferation, rendering them notably susceptible to ferroptosis [30–32]. Consequently, triggering ferroptosis offers a promising strategy for selectively targeting and eliminating tumor cells.

In this study, we demonstrated that depletion of IGF2BP3 induces ferroptosis in glioma. Mechanistically, IGF2BP3 regulates the protein expression level of GPX4 by directly interacting with a specific motif on GPX4 mRNA. Particularly noteworthy, we identified a crucial m6A site on this motif significantly impacting GPX4 mRNA stability and translation. Our study offers insights into IGF2BP3’s mechanistic roles and functional implications, unveiling new possibilities for targeted therapeutic strategies in glioma.

## Results

### Elevated expression of IGF2BP3 is correlated with poor prognosis in TCGA-LGG

**Fig. 1:**
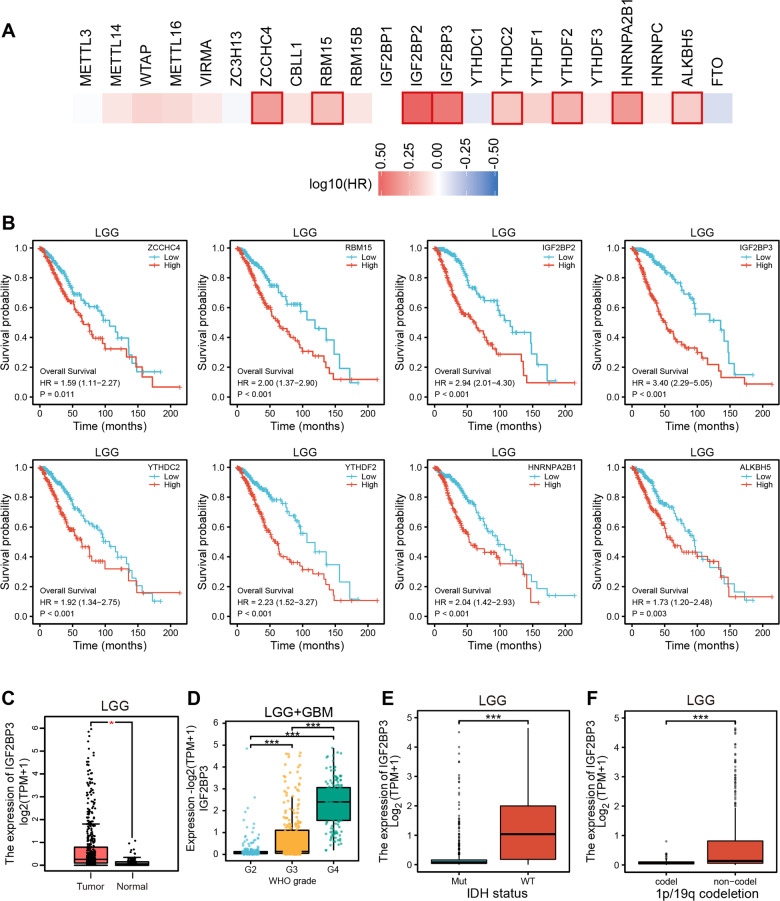
Prognostic significance of m6A regulators in human TCGA-LGG. **A** Survival map showing correlation of expression of 22 m6A regulators with survival time in TCGA-LGG. The red and blue colors indicate poor and favorable prognosis, respectively, with the color intensity corresponding to the magnitude of hazard ratio (HR). A bounding box around tiles shows statistical significance (*p* < 0.05). **B** KM curves showing overall survival for TCGA-LGG patients with high and low expression of ZCCHC4, RBM15, IGF2BP2, IGF2BP3, YTHDC2, YTHDF2, HNRNPA2B1, and ALKBH5. **C** Expression distribution of IGF2BP3 between tumor and normal tissues in TCGA-LGG patients. **D** Expression distribution of IGF2BP3 in different grades of TCGA-LGG and TCGA-GBM patients. **E** Expression distribution of IGF2BP3 in IDH-mutant or IDH-wildtype TCGA-LGG patients. **F** Expression distribution IGF2BP3 in 1p/19q codeletion or 1p/19q non-codeletion TCGA-LGG patients. Statistical significance levels are indicated as follows: *, *p* < 0.05; **, *p* < 0.01; ***, *p* < 0.001.

To investigate the relationship between 22 m6A regulators (Table S1) and their prognostic roles in TCGA-LGG, a survival map (Fig. 1A) was made. This analysis showed that the expression of eight m6A regulators—namely, ZCCHC4, RBM15, IGF2BP2, IGF2BP3, YTHDC2, YTHDF2, HNRNPA2B1, and ALKBH5—was significantly related to poor prognosis in TCGA-LGG (Fig. 1A, B). Among these eight genes, IGF2BP3 showed the highest hazard ratio (HR) value (Fig. 1B, Table S2). Therefore, we subsequently focused on studying the prognostic value of IGF2BP3 in TCGA-LGG. The pan-cancer analysis revealed upregulation of IGF2BP3 expression in a diverse range of human cancers (Fig. S1A). We proceeded to analyze the association between their expression and overall survival across a range of human cancers and found that IGF2BP3 expression was significantly correlated with shorter survival time in many cancer types (Fig. S1B). The expression level of IGF2BP3 exhibits a significant increase in TCGA-LGG or TCGA-GBM cancer tissues compared to normal tissues (Fig. 1C, Fig. S1D). Moreover, its expression is notably elevated in higher grade gliomas (Fig. 1D). The positive correlation between the expression of IGF2BP3 and higher tumor grade was observed in multiple other cancer types (Fig. S1C). The survival analysis revealed no significant association between IGF2BP3 and overall survival in TCGA-GBM patients (Fig. S1E), which indicated IGF2BP3 may be implicated in key malignant transformation step of LGG to GBM. Our further analysis showed that high expression of IGF2BP3 is significantly correlated with higher IDH-wildtype and 1p/19q non-codel status (Fig. 1E, F, Table S3). These results revealed that high IGF2BP3 expression indicates a poor prognosis in LGG.

### Knockdown of IGF2BP3 impairs cell growth and survival in glioma cells

**Fig. 2:**
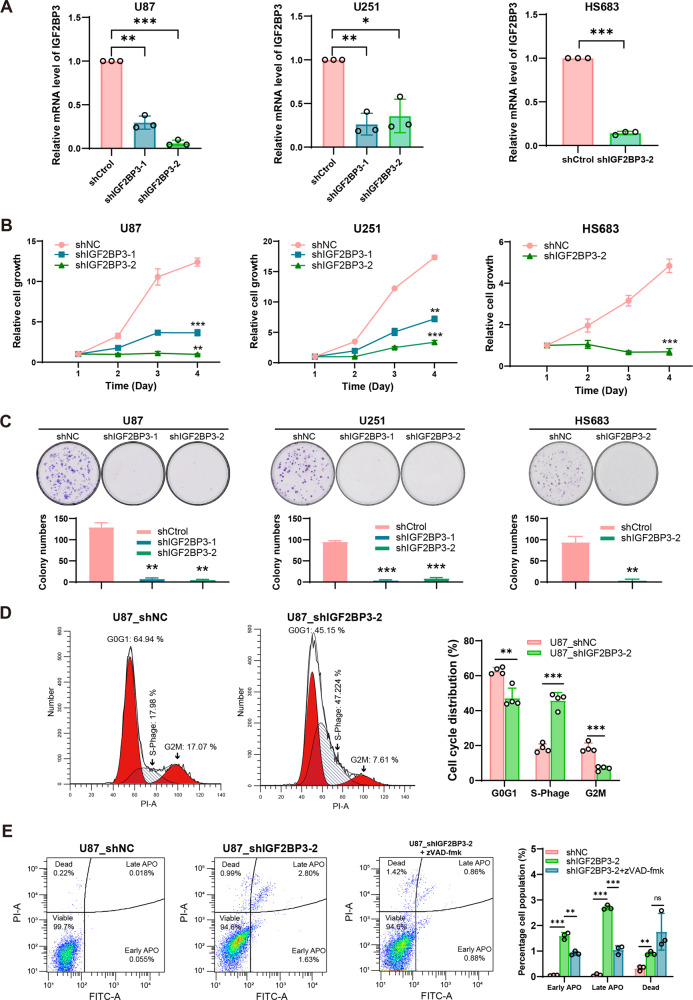
IGF2BP3 impacts on cell viability and proliferation in glioma cells. **A** U87, U251, and HS683 cells were infected with shNC, shIGF2BP3-1, and shIGF2BP3-2 lentivirus. The IGF2BP3 knockdown efficiency was verified by RT-qPCR. Paired two-tailed t-test was used to compare the results (*n* = 3). **B** CCK-8 showing cell viability of U87, U251, and HS683 cells with IGF2BP3 knockdown compared to control cells. Paired two-tailed t-test was used to compare the results (*n* = 3). **C** Colony formation assay showing effects of IGF2BP3 knockdown on clonogenicity in U87, U251, and HS683 cells. Paired two-tailed t-test was used to compare the results (*n* = 3). **D** Cell cycle analysis showing IGF2BP3 knockdown led to S-phase arrest in U87 cells. Multiple t-test was used to compare the results (*n* = 4). **E** Cell apoptosis analysis demonstrating an increase in cell apoptosis and cell death upon IGF2BP3 knockdown in U87 cells. Adding of apoptosis inhibitor zVAD-fmk resulted in partial mitigation of the apoptosis triggered by IGF2BP3 knockdown (Multiple t-test*, n* = 3). Data are presented as the mean ± standard deviation (SD) from three independent experiments. ***p < 0.001, **p < 0.01, *p < 0.05.

To investigate the functional role of IGF2BP3, we knocked down IGF2BP3 in glioma cells, including HS683 (LGG cell line), U87 (GBM cell line), and U251 (GBM cell line). The efficiency of IGF2BP3 mRNA knockdown was validated through RT-qPCR (Fig. 2A). The IGF2BP3-KD cells exhibited a significantly decreased ability in CCK-8 assays (Fig. 2B) and colony formation (Fig. 2C) compared to the control cells, indicating impaired cell viability and clonogenic capacity. Furthermore, to gain insights into the molecular mechanisms underlying the effects of IGF2BP3 knockdown, we performed mRNA sequencing (mRNA-seq) using RNA extracted from U87 IGF2BP3-KD cells and control cells. Differential expression analysis identified a total of 464 up-regulated genes and 521 down-regulated genes in response to IGF2BP3 knockdown (Fig. S2A). To further explore the functional implications of these differentially expressed genes, Gene Ontology (GO) and Gene Set Enrichment Analysis (GSEA) enrichment analyses were conducted. The top four enriched GO terms based on their significance (*p*-value) in the biological process were presented, revealing that the down-regulated genes were primarily associated with mitotic cell division and cell cycle, while the up-regulated genes were mainly enriched in the intrinsic pathway for apoptosis and positive regulation of cell death (Fig. S2B). Consistently, GSEA results highlighted a significant association of IGF2BP3 knockdown with DNA replication and mitotic division (Fig. S2E). These findings were further supported by cell cycle analysis, demonstrating that IGF2BP3 knockdown induced a distinct cell cycle disruption characterized by S-phase arrest (Fig. 2D, Fig. S2C). Furthermore, the analysis of cell apoptosis revealed a significant increase in cellular apoptosis and death upon IGF2BP3 knockdown (Fig. 2E, Fig. S2D). Intriguingly, the introduction of the pan-caspase apoptosis inhibitor zVAD-fmk led to a partial mitigation of the observed apoptotic response or cell death outcome (Fig. 2E, Fig. S2D), suggesting that the induction of cell apoptosis is partial mediated through caspase-dependent pathways. However, it appears that cell death may also be regulated by caspase-independent mechanisms. Additionally, GSEA indicated that IGF2BP3 may play a role in ferroptosis, as it showed a correlation with oxidative phosphorylation and mitochondrial electron transport cytochrome c to oxygen (Fig. S2E).

### IGF2BP3 knockdown induces ferroptosis in glioma cells

**Fig. 3:**
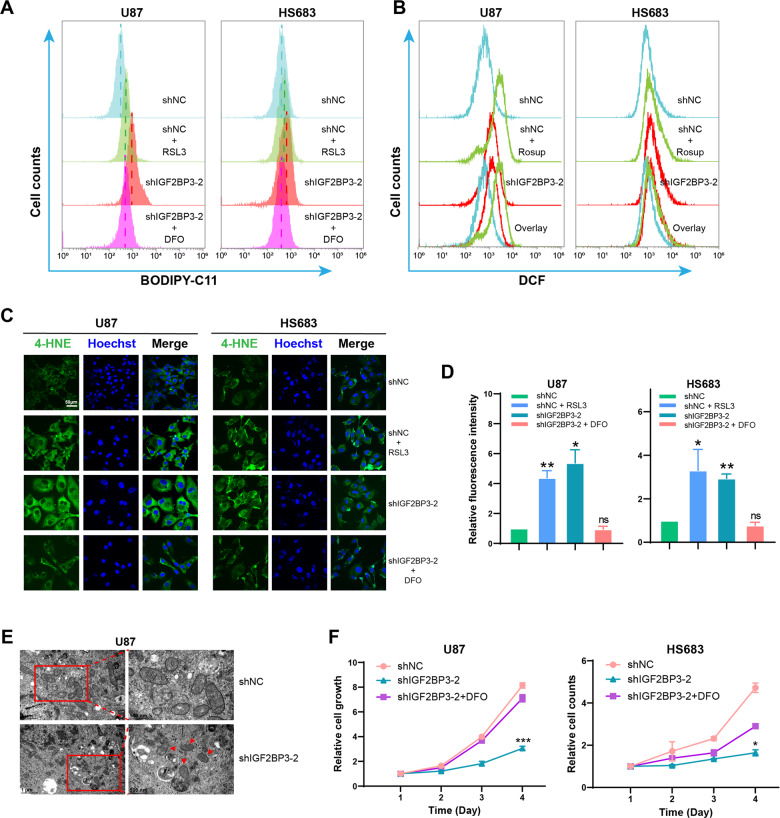
IGF2BP3 knockdown induces ferroptosis in glioma cells. Flow cytometry histograms show **A** the levels of lipid peroxides assessed using BODIPY-C11 probe staining, and **B** ROS levels measured by DCF probe in IGF2BP3-KD U87 and HS683 cells compared to control cells. Cells were treated RSL3 to induce ferroptosis. **C** Immunofluorescence images showing 4-HNE expression in IGF2BP3-KD U87 and HS683 cells compared with control cells. **D** The fluorescent intensity in (**C**) was quantified using Image Pro-Plus software, and the data were analyzed using paired two-tailed t-test (*n* = 3). **E** Transmission electron microscopy images revealed that IGF2BP3-KD U87 cells exhibited smaller mitochondria with heightened membrane density and decreased mitochondrial cristae (indicated by red arrows). **F** CCK-8 analysis showing DFO treatment rescue cell viability in IGF2BP3-KD U87 and HS683 cells (multiple t-tests). Data are presented as the mean ± standard deviation (SD) from three independent experiments. ***p < 0.001, **p < 0.01, *p < 0.05.

Upon IGF2BP3 knockdown in U87 cells, we observed distinctive morphological changes characterized by cellular swelling (Fig. S3A), which is a typical phenotype observed during ferroptosis [33]. To further investigate the potential involvement of ferroptosis, we performed BODIPY-C11 staining followed by flow cytometry analysis and immunofluorescent staining to detect 4-hydroxynonenal (4-HNE), a marker of lipid peroxidation. Our results from BODIPY-C11 staining (Fig. 3A) and immunofluorescence analyses (Fig. 3C, D) demonstrated a significant increase in lipid peroxidation levels in IGF2BP3-KD U87 and HS683 cells compared to control cells. Moreover, the addition of deferoxamine (DFO), a ferroptosis inhibitor, to IGF2BP3-KD cells reduced the lipid peroxidation level (Fig. 3A, C, and D), suggesting that the observed increase in lipid peroxidation is associated with ferroptosis. To evaluate the levels of reactive oxygen species (ROS) in the cells, we utilized the H2DCFDA Reactive Oxygen Species Assay Kit followed by flow cytometry and microscopy. The flow cytometry results (Fig. 3B) and microscopy images (Fig. S3B) consistently demonstrated elevated levels of ROS in IGF2BP3-KD cells compared to the control cells. To confirm the ultrastructural changes associated with ferroptosis, transmission electron microscopy (TEM) was performed, which showed smaller mitochondria with increased membrane density and reduced mitochondrial cristae in IGF2BP3-KD U87 cells compared to control cells (Fig. 3E). The CCK-8 assay results showed that the addition of DFO to IGF2BP3-KD U87 and HS683 cells significantly rescued cell viability (Fig. 3F), indicated that the impaired cell viability in IGF2BP3-KD cells is induced by ferroptosis. Taken together, these findings suggest that IGF2BP3 knockdown induces ferroptosis in glioma cells.

### IGF2BP3 positively regulates GPX4 protein expression in glioma

**Fig. 4:**
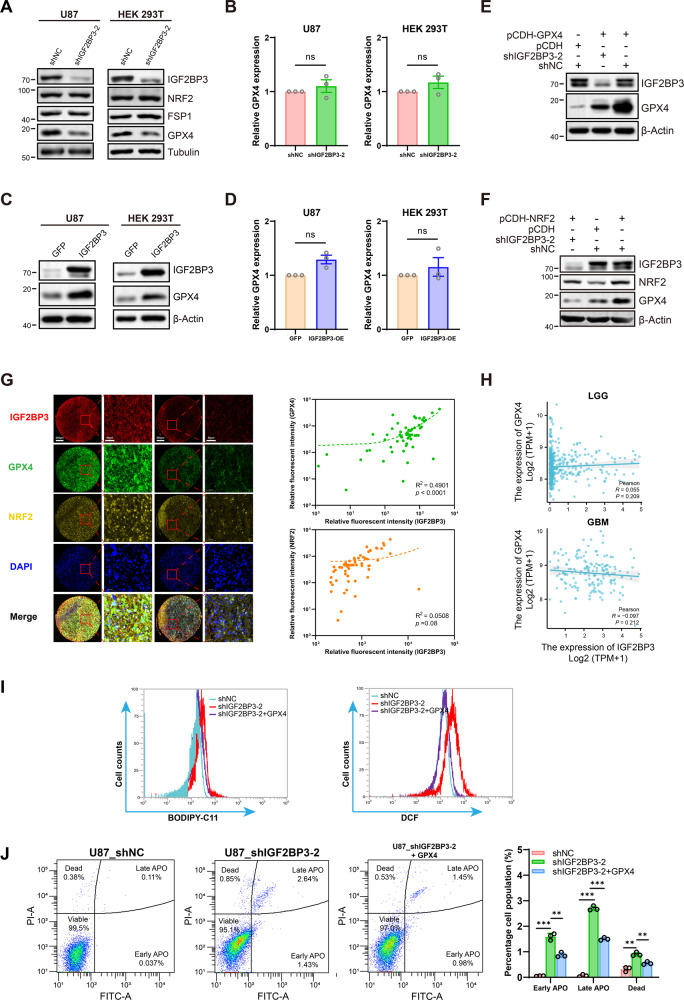
Correlation of IGF2BP3 and GPX4 expression. **A** Western blot detecting NRF2, FSP1, and GPX4 protein levels in IGF2BP3-KD U87 and HEK 293 T cells compared with control cells. **B** RT-qPCR analysis detecting GPX4 mRNA level in IGF2BP3-KD U87 and HEK 293 T cells compared with control cells (Paired two-tailed t-test). **C**-**D** The GPX4 protein expression level was verified by western blot (**C**) and mRNA expression level was verified by RT-qPCR (**D**) in IGF2BP3 overexpressing U87 and HEK 293 T cells compared with GFP control cells (Paired two-tailed t-test). **E** U87 cells were infected with shIGF2BP3-2 lentivirus to knockdown IGF2BP3 or infected with shNC lentivirus as a control. Subsequently, cells were infected with pCHD-GPX4 to overexpress GPX4. The GPX4 protein expression level was verified by western blot. **F** U87 cells were infected with shIGF2BP3-2 lentivirus to knockdown IGF2BP3 or infected with shNC lentivirus as a control. Subsequently, cells were infected with pCDH-NRF2 to overexpress NRF2 or infected with pCDH empty vector as a control. The GPX4 protein expression level was verified by western blot. **G** Tissue microarray chip containing clinical glioma patients’ tumor samples (*n* = 60) was analyzed using mIHC to detect the protein expression levels of IGF2BP3, NRF2, and GPX4. The expression correlation between IGF2BP3 and GPX4 or NRF2 was fitted using a linear regression approach. **H** Correlation between IGF2BP3 and GPX4 mRNA expression levels in TCGA-LGG and TCGA-GBM samples. Pearson correlation was conducted to analyze the correlation. **I** U87 cells were infected with shIGF2BP3-2 lentivirus to knockdown IGF2BP3 or infected with shNC lentivirus as a control. Subsequently, cells were infected with pCHD-GPX4 to overexpress GPX4. The levels of lipid peroxides were assessed using BODIPY-C11 probe staining, and ROS levels was measured by DCF probe. The results were presented as flow cytometry histograms. **J** Cell apoptosis analysis demonstrating GPX4 overexpression partial rescued the cell apoptosis and cell death induced by IGF2BP3 knockdown in U87 cells (multiple t-tests). Data are presented as the mean ± standard deviation (SD) from three independent experiments. ***p < 0.001, **p < 0.01, *p < 0.05.

Increased lipid peroxidation, elevated ROS levels, and ultrastructural modifications in mitochondria suggest that IGF2BP3 may play a critical role in regulating ferroptosis in glioma cells. The mechanistic details underlying the relationship between IGF2BP3 and ferroptosis regulation remain to be elucidated. GPX4 serves as a key regulator of ferroptosis by reducing lipid hydroperoxides to non-toxic lipid alcohols [29, 34]. Additionally, ferroptosis suppressor protein 1 (FSP1) has emerged as a crucial player in the regulation of ferroptosis [35, 36]. The nuclear factor erythroid 2-related factor 2 (NRF2) is a pivotal transcription factor that plays a crucial role in ferroptosis [37, 38]. Therefore, we performed a western blot assay to examine the expression levels of GPX4, NRF2, and FSP1 subsequent IGF2BP3 knockdown. The results revealed a decrease in the expression of GPX4 upon IGF2BP3 knockdown, while FSP1 and NRF2 expression remained unchanged (Fig. 4A). Notably, the observed decrease in GPX4 protein levels following IGF2BP3 knockdown did not coincide with a decrease in GPX4 mRNA levels (Fig. 4B). Overexpression of IGF2BP3 resulted in increased GPX4 protein levels (Fig. 4C) without affecting GPX4 mRNA levels (Fig. 4D). Furthermore, when GPX4 was overexpressed in both IGF2BP3-KD and control U87 cells, the GPX4 protein expression in IGF2BP3-KD U87 cells was notably lower than in control cells (Fig. 4E). These findings suggest that IGF2BP3 likely exerts a regulatory function over GPX4 in a post-transcriptional manner. Previous study has indicated that IGF2BP3 has the capability to bind to NRF2 mRNA, thereby influencing its mRNA stability [39]. Additionally, GPX4 has been identified as a downstream target of NRF2 [37, 38]. To gain further insights into whether IGF2BP3 regulates GPX4 expression directly or through the intermediation of NRF2, we conducted experiments involving the overexpression of NRF2 in U87 cells with or without IGF2BP3 knockdown. The results demonstrated that NRF2 overexpression led to elevated GPX4 expression in U87 control cells (Fig. 4F). However, even in the presence of NRF2 overexpression, the GPX4 expression level in IGF2BP3-KD cells remained comparatively lower than that observed in control cells (Fig. 4F). To further explore the expression relationship between IGF2BP3, GPX4, and NRF2, a multi-color immunohistochemistry (mIHC) assay was conducted using a glioma tumor tissue microarray (TMA) obtained from 60 clinical glioma patients’ samples. The results revealed a significant positive correlation between IGF2BP3 and GPX4 protein expression, while the correlation between IGF2BP3 and NRF2 was not significant (Fig. 4G and Fig. S4). Further analysis of the correlation between GPX4 and IGF2BP3 mRNA within the TCGA-LGG and TCGA-GBM samples did not reveal any significant association (Fig. 4H). These findings collectively suggest the potential for IGF2BP3 to exert a direct regulatory influence on GPX4 protein expression levels, without significantly affecting its mRNA expression. The restoration of GPX4 expression in IGF2BP3-KD U87 cells resulted in a reduction of lipid peroxide and ROS levels (Fig. 4I) induced by IGF2BP3 knockdown. Furthermore, this rescue of GPX4 expression led to a partial mitigation of the cell apoptosis and cell death provoked by IGF2BP3 knockdown (Fig. 4J), indicating that the diminished GPX4 levels might initiate the apoptosis triggered by IGF2BP3 knockdown, as the reduction in GPX4 levels, resulting in oxidative damage, could potentially act as an apoptosis-inducing signal [40]. These outcomes provide additional substantiation for the hypothesis that the induction of ferroptosis following IGF2BP3 knockdown is likely mediated through its regulatory influence on GPX4.

### The specific m6A modification site on GPX4 mRNA binds to IGF2BP3 and plays a crucial role in regulating GPX4 expression

**Fig. 5:**
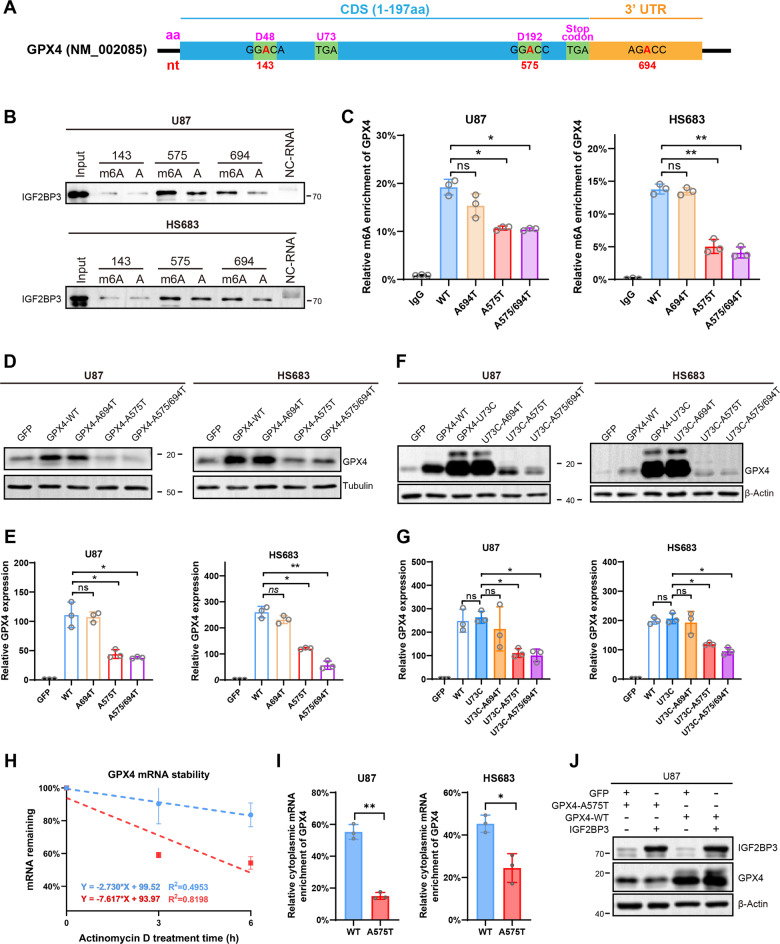
The nucleotide A575 in GPX4 (NM_002085) transcript is critical for GPX4 protein expression. **A** Cartoon diagram illustrating the Coding Sequence (CDS) and 3’UTR of GPX4 (NM_002085) transcript. The amino acid numbering was highlighted in magenta, and the nucleotide numbering was highlighted in red. Three potential IGF2BP3 binding sequences and the UGA codons are indicated in the diagram. **B** RNA pull-down assay to assess the binding potency of three RNA sequences with IGF2BP3. Three potential IGF2BP3 binding RNA sequences were synthesized with or without m6A modification and then mixed with lysate from U87 and HS683 cells. The RNA-protein complexes were pulled down, and an anti-IGF2BP3 western blot was performed to detect the interaction between IGF2BP3 and the RNA sequences. **C** MeRIP assay revealing m6A enrichment of different GPX4 mutants. U87 and HS683 cells were infected with lentivirus overexpressing GPX4-WT, GPX4-A575T, GPX4-A694T, or GPX4-A575/694 T. Total RNA was extracted, and mRNA was purified. The mRNA was then enriched using an anti-m6A antibody and quantified by qPCR (Paired two-tailed t-test). **D**-**E** The U87 and HS683 cells were infected with lentivirus overexpressing GPX4-WT, GPX4-A575T, GPX4-A694T, or GPX4-A575/694T. The GPX4 protein and mRNA expression levels were verified by western blot (**D**) and qPCR (**E**). **F**-**G** The U87 and HS683 cells were infected with lentivirus overexpressing GPX4-WT, GPX4-U73C, GPX4-U73C-A575T, GPX4-U73C-A694T, or GPX4-U73C-A575/694T. The GPX4 protein and mRNA expression levels were verified by western blot (**F**) and qPCR (**G**). **H** HEK 293 T cells were infected with lentivirus overexpressing GPX4-WT or GPX4-A575T, and treated with Actinomycin D for 0, 3, and 6 h. RNA was extracted, and qPCR was performed to verify the expression level of GPX4. The degradation lines were generated using linear regression approach. **I** The U87 and HS683 were infected with lentivirus overexpressing GPX4-WT or GPX4-A575T. Cytoplasmic and whole cells’ RNA was extracted, and the expression rate of GPX4 in the cytoplasm relative to the whole cells was verified using qPCR. **J** IGF2BP3 was overexpressed together with GPX4-WT or GPX4-A575T, the expression level of GPX4 was verified by western blot. Data are presented as the mean ± standard deviation (SD) from three independent experiments. ***p < 0.001, **p < 0.01, *p < 0.05.

The obtained results suggested that IGF2BP3 potentially exerts a directed regulatory function on GPX4, possibly by directly binding to specific m6A-modified sites on GPX4 mRNA. Three potential m6A modification sites were predicted within the coding sequence (CDS) and 3’ untranslated region (UTR) of GPX4 mRNA (Fig. S5A). Importantly, these three sites exhibited sequence conservation and remained identical across all four GPX4 transcripts (Fig. S5A). For clarity, we used the NM_002085 numbering system in the present study (Fig. 5A).

To validate the binding potential of IGF2BP3 to these predicted m6A-modified sites, RNA pull-down assays were performed using synthesized RNA with or without m6A modification. The results showed that the GGA_575_CC and AGA_694_CC sequences with m6A modification pulled down more IGF2BP3 from U87 or HS683 cell lysates compared to those without m6A modification (Fig. 5B). This suggests that GG(m6A_575_)CC and AG(m6A_694_)CC on GPX4 mRNA are potential binding sites for IGF2BP3. To confirm the occurrence of m6A modification at these two adenine sites in cells, adenine (A) was substituted with thymine (T), and a Methylated (m6A) RNA Immunoprecipitation (MeRIP) assay was conducted. U87 and HS683 cells overexpressing GPX4-WT, GPX4-A575T, GPX4-A694T, or GPX4-A575/694 T were generated using a lentiviral system. Subsequently, mRNA was extracted and immunoprecipitated using an anti-m6A antibody, followed by RT-qPCR analysis. The results demonstrated that the enrichment of GPX4-A575T mRNA by the m6A antibody was significantly lower than that of GPX4-WT, while GPX4-A694T did not show significant changes (Fig. 5C). The m6A enrichment of double mutant GPX4-A575/694 T showed a similar level to that of the GPX4-A575T mutant (Fig. 5C), indicating that A575 is a pivotal site undergoing m6A modification in cells.

To assess the biological significance of m6A modification at these two sites, GPX4-WT or mutants were overexpressed in U87, HS683, and HEK 293 T cells, and the protein and mRNA expression levels of GPX4 were measured. Western blot and qPCR analyses revealed that the protein and mRNA expression levels of GPX4-A575T were significantly lower than those of GPX4-WT, while the GPX4-A694T mutant showed no significant changes compared to GPX4-WT, and the double mutant GPX4-A575/694 T showed similar expression pattern to that of GPX4-A575T (Fig. 5D and E, Fig. S5B and C). These results suggest that A575 on GPX4 mRNA is critical for its expression. Notably, despite the much higher mRNA expression level of GPX4-A575T compared to the GFP control, the protein level of GPX4-A575T was similar to that of the GFP control (Fig. 5D and E, Fig. S5B and C). This suggests that m6A modification at A575 may have a substantial impact not only on GPX4 mRNA expression but also on the translation of mRNA into protein. As a selenoprotein, GPX4 contains selenocysteine (Sec, U) inserted within its coding sequence (Fig. 5A). Selenocysteine is encoded by the UGA codon, which normally functions as a stop codon. Its translation requires the presence of a selenocysteine insertion sequence (SECIS) element within the mRNA structure. To investigate whether the effects of m6A modification at A575 on GPX4 expression are related to selenocysteine translation, a mutation was introduced to replace selenocysteine (U) with cysteine (C). The results of western blot and qPCR analyses demonstrated that the expression of GPX4-U73C-A575T was significantly lower than that of GPX4-U73C (Fig. 5F and G, Fig. S5D and E), indicating that the effects of A575 on GPX4 expression are not attributed to selenocysteine translation.

To gain further insight into the regulatory mechanism underlying GPX4 expression by A575, we conducted experiments to examine mRNA stability and subcellular localization of GPX4-A575T compared to GPX4-WT. The results revealed that GPX4-A575T exhibited a faster degradation rate with a shorter half-life compared to GPX4-WT (Fig. 5H), indicating that m6A modification at A575 plays a role in maintaining mRNA stability. The process of mRNA translation into protein predominantly takes place within the cytoplasm. To assess the translocation of mRNA, RNA was extracted from the cytoplasmic fraction, followed by qPCR analysis. The efficiency of cytoplasmic fraction isolation was validated by western blot (Fig. S5G). The findings demonstrated a significant reduction in the cytoplasmic enrichment of GPX4-A575T compared to GPX4-WT (Fig. 5I), indicating that m6A modification at A575 may play a crucial role in facilitating the transport of GPX4 mRNA to the cytoplasm. Notably, IGF2BP3 knockdown had no significant influence on the accumulation of GPX4 mRNA in the cytoplasm (Fig. S5F), indicating that the regulatory function of IGF2BP3 towards GPX4 expression is not attributed to its impact on mRNA subcellular localization. To explore whether the regulatory function of IGF2BP3 towards GPX4 protein expression is mediated through binding to the GG(m6A_575_)CC sequence on GPX4, GPX4-A575T or GPX4-WT was co-expressed with IGF2BP3. Western blot analysis revealed that when co-expressed with IGF2BP3, the expression level of GPX4-WT was significantly higher compared to the GFP control, whereas GPX4-A575T co-expressed with IGF2BP3 showed no changes compared to the GFP control (Fig. 5J). These findings strongly suggest that IGF2BP3 regulates GPX4 expression by specifically binding to the GG(m6A_575_)CC sequence on GPX4 mRNA.

In summary, these findings suggest that IGF2BP3 have a directed regulatory role in GPX4 through direct binding to the m6A-modified site on GPX4 mRNA. The m6A modification at A575 plays a crucial role in regulating GPX4 expression at both the mRNA and protein levels.

### IGF2BP3 is a promising therapeutic target for glioma

**Fig. 6:**
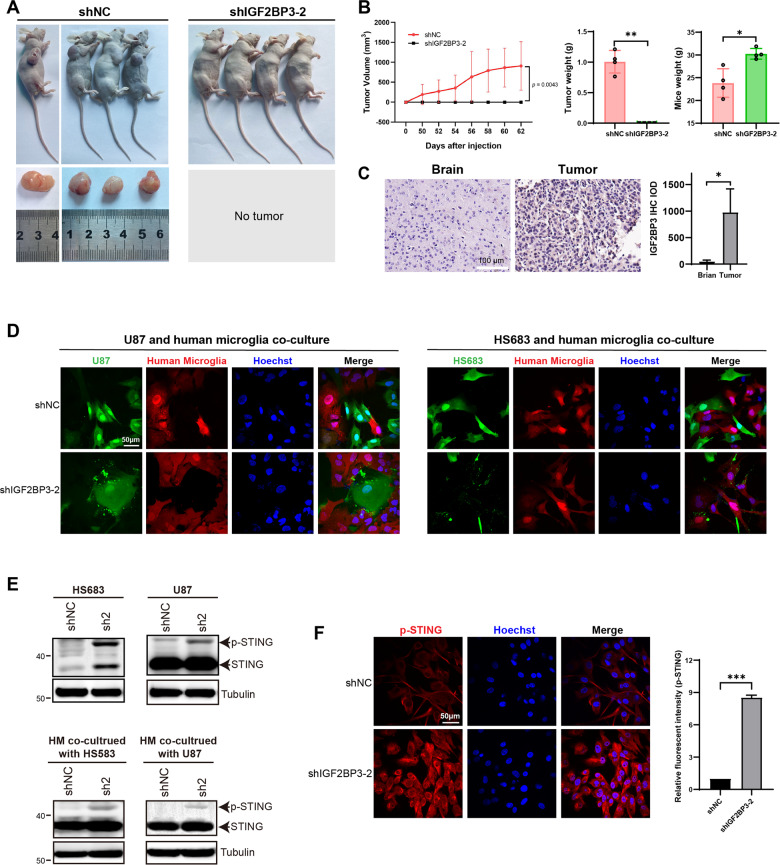
IGF2BP3 knockdown glioma cells fail to form tumors in xenograft mice model and are more susceptible to human microglia cells. **A** 5 ×10^6^ U87 cells with or without IGF2BP3 knockdown were subcutaneously injected into the rear flank of nude mice. Mice were sacrificed, and the tumors were excised (*n* = 4). **B** The volume of tumors was measured every two days before mice were sacrificed, tumor volume was calculated using the formula V = (W^2^ × L) / 2, where V represents the tumor volume, W indicates the tumor width, and L denotes the tumor length; the weight of the tumors and mice was measured after mice were sacrificed (*n* = 4). **C** The expression of IGF2BP3 in xenograft tumors and normal mice brains was assessed using IHC. The differences in IGF2BP3 expression between tumors and brains were quantified using Image Pro-Plus software and analyzed using paired two-tailed t-test (*n* = 4). **D** U87-GFP and HS683-GFP cells with or without IGF2BP3 knockdown were co-cultured with human microglia-mCherry cells. The phagocytic function of microglia cells was visualized using confocal microscopy. **E** U87 and HS683 cells were infected with shIGF2BP3-2 lentivirus to knockdown IGF2BP3 or infected with shNC lentivirus as a control. The protein expression levels of STING and p-STING in U87 and HS683 cells, as well as in human microglia cells co-cultured with U87 and HS683 cells, were verified using western blot analysis. **F** The p-STING expression level in HS683 cells with or without IGF2BP3 knockdown was assessed using immunofluorescence. The fluorescent intensity was quantified using Image Pro-Plus software, and the data were analyzed using paired two-tailed t-test. Data are presented as the mean ± standard deviation (SD) from three independent experiments. ***p < 0.001, **p < 0.01, *p < 0.05.

To assess the functional effects of IGF2BP3 in glioma tumor formation, a xenograft assay was performed. Strikingly, IGF2BP3-KD U87 cells failed to form tumors and the IGF2BP3-KD group of mice exhibited significantly higher body weight compared to the control group (Fig. 6A, B), indicating a critical role of IGF2BP3 in glioma tumor formation. IHC analysis revealed that IGF2BP3 expression was barely detectable in normal mouse brain tissue, while it was prominently expressed in xenograft glioma tumors (Fig. 6C). Co-culture of glioma cells with human microglia (HM) cells demonstrated that IGF2BP3-KD U87 and HS683 cells were more susceptible to phagocytosis by microglia (Fig. 6D). Western blot and immunofluorescence assays revealed that IGF2BP3-KD U87 and HS683 cells displayed increased levels of p-STING (Fig. 6E, F). Consistently, human microglia (HM) cells co-cultured with IGF2BP3-KD U87 and HS683 cells also exhibited elevated p-STING levels compared to those co-cultured with control U87 and HS683 cells (Fig. 6E), indicating the potential triggering of STING activation in both glioma cells and microglia upon IGF2BP3 knockdown. To gain further insights into the tissue-specific expression pattern of IGF2BP3, we analyzed its mRNA expression level in various human organs using the HPA, FANTOM5, and GTEx databases. The collective analysis revealed that IGF2BP3 mRNA expression was predominantly restricted to placenta, thymus, and testis, with minimal expression observed in other organs (Fig. S6). Furthermore, the pan-cancer analysis demonstrated significant overexpression of IGF2BP3 in most cancer types (Fig. S1A), indicating its potential as a promising therapeutic target not only for glioma but also for other solid tumors.

**Fig. 7:**
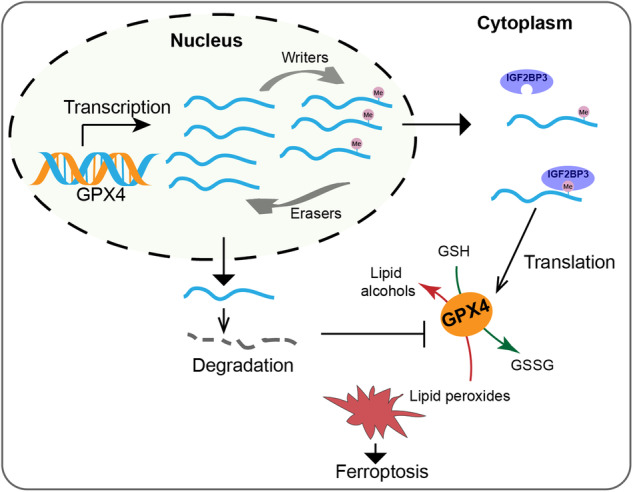
Graphic diagram illustrating the importance of GPX4 mRNA binding to IGF2BP3 and its m6A modification for GPX4 protein expression. After transcription, the level of m6A in GPX4 mRNA is dynamically modulated by regulators. GPX4 mRNA with m6A modification on A575 site is prone to cytoplasmic transportation and can bind to IGF2BP3, facilitating its translation into protein. In contrast, GPX4 mRNA without m6A modification is more susceptible to degradation. Reduced GPX4 protein expression leads to the accumulation of lipid peroxidation, resulting in ferroptosis.

In summary, our findings collectively demonstrate that IGF2BP3 plays a pivotal role in modulating GPX4 by directly interacting with GPX4 mRNA, thereby orchestrating the regulation of ferroptosis in glioma. Importantly, the m6A modification at the specific site on GPX4 mRNA emerges as a critical determinant for its mRNA stability and translation (Fig. 7).

## Discussion

Increasing evidence suggests that IGF2BP3 is frequently overexpressed and associated with unfavorable prognosis in various cancer types [41–43], including glioma [24, 39, 44, 45]. While previous studies have highlighted IGF2BP3’s role in promoting cell proliferation, migration, and invasion in glioma [45–48], the specific targets and the intricate mechanisms through which IGF2BP3 regulates cell growth and death remain elusive. The identified enrichment of IGF2BP3-regulated genes in cell cycle-related and apoptosis-related pathways in this study further underscores its role in cell growth control. This observation is consistent with previous findings from global gene expression profiling studies in U251 cells [49]. The study provides a perspective on how IGF2BP3 is engaged in regulating of cancer growth and survival. Current evidence suggests that IGF2BP3 modulates target gene expression by influencing RNA stability or translation [50, 51]. Whether IGF2BP3 regulates RNA stability or translation of cell growth and death targets warrants further investigation.

Additionally, our investigation utilizing the HPA, FANTOM5, and GTEx databases unveiled that IGF2BP3 mRNA is discernible in a limited range of human organs. Notably, consistent with our mRNA analysis, immunohistochemical analyses of normal human tissue samples confirmed that IGF2BP3 protein expression is detectable exclusively in placenta, lymph nodes, tonsils, and testes [44]. This unique expression pattern could serve as a foundation for the development of therapies that selectively target cancer cells while avoiding impacting healthy tissues.

The diverse attributes characterizing IGF2BP3, such as its widespread overexpression in various cancer types, its regulatory control over genes influencing cell growth, and its restricted presence in specific human organs, collectively position it as a prime candidate for cancer therapy.

A particularly notable finding of this study is the identification of a specific m6A modification site (GGA_575_CC) on GPX4 mRNA that holds crucial significance for GPX4 expression. When the A_575_ is mutated to T, the exogenous GPX4 protein expression becomes significantly diminished, despite the mRNA level remaining notably high. This observation highlights the indispensable role of this specific m6A site in GPX4 mRNA translation to protein. By targeting this site, either on DNA or on mRNA, researchers can potentially modulate GPX4 expression, thereby influencing ferroptosis susceptibility. Our results make it possible to alter the expression level of GPX4 by changing only one base sequence. Adenine base editors (ABEs), which is capable of converting adenine (A) to guanine (G) mutations [52], holds the promise of fine-tuning GPX4 expression levels, may offer the possibility of fine-tuning GPX4 levels. In addition to DNA editing, the application of A-to-I RNA base editors (xABEs) [53], may also provide a powerful tool for manipulating GPX4 expression. The ability to modify a single base sequence and thereby influence GPX4 expression levels presents an intriguing potential for a therapeutic strategy.

While our study has advanced our understanding of the intricate interplay between IGF2BP3, GPX4, and ferroptosis regulation in glioma, it has also surfaced intriguing questions that warrant further exploration. One critical avenue for future exploration lies in deciphering the precise mechanism through which IGF2BP3 exerts its influence on translation. Understanding the intricate interplay between IGF2BP3 and translation machinery could provide a deeper understanding of the regulatory processes underlying the observed effects on GPX4 and ferroptosis. Additionally, the identification of a single base pair within the GPX4 transcript that exerts substantial influence on its expression raises compelling questions surrounding the exact molecular basis of this regulatory interaction. Understanding how such single base pair variation can wield significant effects on gene expression has the potential to uncover novel regulatory paradigms.

## Materials and Methods

### TCGA database analysis

The survival map and Kaplan–Meier (KM) curves were made using the GEPIA 2 (http://gepia2.cancer-pku.cn/#survival) or UALCAN (http://ualcan.path.uab.edu/analysis.html) online tools. Overall survival was selected as the survival method; the median threshold was selected to distinguish the high- and low-expression cohorts; the log-rank test was used for statistical analysis, with *p* < 0.05 considered to indicate a statistically significant difference; and HR values were calculated using the Cox proportional hazards model. The association between IGF2BP3 expression and overall survival across human cancers was analyzed using the TISIDB online tool (http://cis.hku.hk/TISIDB/index.php).

Gene expression profiles and clinical information were downloaded from GDC (https://portal.gdc.cancer.gov/) in level 3 HTSeq-FPKM format. FPKM (fragments per kilobase per million) values were converted to TPM (transcripts per million reads). The median threshold was selected to split the data into high- and low-expression cohorts. The Shapiro–Wilk W-test and the Wilcoxon rank sum test were used for statistical analysis, with *p* < 0.05 again considered to indicate statistical significance. Associations were analyzed using Pearson correlation and quantified by Pearson’s correlation coefficient (r), taking *p* < 0.05 to represent statistical significance.

Data for WHO grade, IDH status, and 1p/19q codeletion status were obtained from the study conducted by Ceccarelli et al. [5]. The median threshold was selected to split the data into high- and low-expression cohorts. The Shapiro–Wilk normality test and the Wilcoxon rank sum test were used for statistical analysis, with *p* < 0.05 again considered to indicate statistical significance.

### Mammalian cell culture

U87, HS683, U251, HEK 293 T, and human microglia cells were cultured in DMEM medium supplemented with 10% fetal bovine serum, 2 mM glutamine, and 100 U/mL penicillin/streptomycin at 37 °C with 5% CO_2_. All human cell lines have been recently authenticated by STR profiling and tested for mycoplasma contamination.

### Lentiviral infection

For knockdown of IGF2BP3, two shRNA sequences were used: shIGF2BP3-1 (5’_CCGGGCAGGAATTGACGCTGTATAACTCGAGTTATACAGCGTCAATTCCTGCTTTTTG_3’) and shIGF2BP3-2 (5’_CCGGTGTTGTAGTCTCACAGTATAACTCGAGTTATACTGTGAGACTACAACATTTTTG_3’). A non-specific control RNA sequence was used as a control: 5’_GTTCTCCGAACGTGTCACGTCTCGAGACGTGACACGTTCGGAGAACTTTTT_3’. To produce lentiviruses, the pLKO.1 vector containing the respective shRNA or shNC RNA was co-transfected with pCMV-dR8.2 dvpr and pCMV-VSV-G into HEK 293 T cells.

To overexpress IGF2BP3 (NM_006547), GPX4 (NM_002085), GPX4 mutants, NRF2 (NM_006164), and GFP, their respective coding sequences were cloned into the pCDH-CMV-MCS-EF1-Puro vector. For lentiviral production, the overexpressing vector containing the gene of interest was co-transfected with pCMV-dR8.2 dvpr and pCMV-VSV-G into HEK 293 T cells.

After 40 h of transfection, the lentivirus was harvested and filtered through a 0.45 μm filter. Subsequently, the lentivirus was added to the target cells along with 10 μg/mL polybrene. After two days of infection, cells were subjected to selection using 2.5 μg/mL puromycin.

### RNA isolation and real-time quantitative PCR (qPCR)

Total RNA was isolated from the cells using the RNAeasy™ Animal RNA Isolation Kit (Beyotime, Cat# R0027, China) according to the manufacturer’s instructions. To synthesize cDNA, 1 μg of the isolated total RNA was used as a template in a reverse transcription reaction. The HiScript Q RT SuperMix for qPCR kit (Vazyme, Cat# R223, China) was utilized following the manufacturer’s protocol. Real-time quantitative PCR (qPCR) was performed using the ChamQ SYBR Color qPCR Master Mix (Vazyme, Cat# Q321, China) in accordance with the manufacturer’s instructions. The relative mRNA expression levels were determined using the 2^−ΔΔCt^ method. The primer sequences used were as follows:

GPX4-Forward: CGGAATTCATGAGCCTCGGCCGCCTTTG;

GPX4-Reverse: CCGCTCGAGGAAATAGTGGGGCAGGTCCT;

GAPDH-Forward: GTCTCCTCTGACTTCAACAGCG;

GAPDH-Reverse: ACCACCCTGTTGCTGTAGCCAA.

### Cell viability assay

Cell viability was tested by a Cell Counting Kit-8 kit (Beyotime, Cat# C0038, China). Cells were seeded into 96-well plates at a density of 2000 cells/well. CCK8 reagent was added to cells and incubated at 37°C for 1 h. Following the incubation, the absorbance of the formazan product was measured at 450 nm using a microplate reader.

### Colony formation assay

U87, HS683, and U251 cells with IGF2BP3 knockdown, as well as control cells, were seeded in 6-well plates at a density of 200 cells/mL. The cells were then cultured in a 37 °C incubator for 1–2 weeks until the control cells formed visibly large colonies. To assess colony formation, cells were fixed with 4% paraformaldehyde and stained with 0.5% crystal violet in 20% ethanol for 30 minutes. Subsequently, the plates were gently washed with tap water, allowed to dry at room temperature, and the colonies were visually counted.

### mRNA sequencing

Total RNA was isolated using the RNAeasy™ Animal RNA Isolation Kit (Beyotime, Cat# R0027, China) following the manufacturer’s instructions. The quality and quantity of the total RNA were assessed using the Bioanalyzer 2100 and RNA 6000 Nano LabChip Kit (Agilent, Cat# 5067-1511, USA). High-quality RNA samples with a RIN (RNA Integrity Number) greater than 7.0 were selected for library construction. To enrich for mRNA, Dynabeads Oligo (dT) (Thermo Fisher, USA) was used for purification. The purified mRNA was then fragmented into short fragments. Subsequently, the cleaved RNA fragments were reverse transcribed to synthesize cDNA using SuperScript™ II Reverse Transcriptase (Invitrogen, Cat# 1896649, USA). The constructed cDNA libraries were subjected to 2 × 150 bp paired-end sequencing (PE150) on an Illumina Novaseq™ 6000 (LC-Bio Technology CO., Ltd., Hangzhou, China) following the vendor’s recommended protocol. For gene expression analysis, DESeq2 software was used to compare gene expression levels between two different groups, and edgeR was used to compare gene expression between two samples. Genes with a false discovery rate (FDR) below 0.05 and an absolute fold change of at least 2 were considered as differentially expressed genes (DEGs).

### Gene ontology (GO) enrichment analysis and gene set enrichment analysis (GSEA)

All DEGs were mapped to GO terms in the Gene Ontology database (http://www.geneontology.org/), gene numbers were calculated for every term, significantly enriched GO terms in DEGs comparing to the genome background were defined by hypergeometric test. GO terms with a p-value less than 0.05 are considered significantly enriched among the DEGs.

Gene set enrichment analysis was performed using software GSEA (v4.1.0) and MSigDB to identify whether a set of genes in specific GO terms shows significant differences in two groups. The genes in the dataset are ranked based on their differential expression between two groups, using Signal2Noise normalization method. Enrichment scores and *p* value was calculated in default parameters.

### Cell cycle analysis

IGF2BP3 knockdown or control U87 and HEK 293 T cells were seeded to 6-well plates at an appropriate density. Subsequently, the cells were trypsinized, washed with PBS, and fixed in 70% ethanol at 4 °C overnight. The fixed cells were then collected and washed with PBS to remove any residual ethanol. The cells were stained with Propidium Iodide (PI) using the Cell Cycle and Apoptosis Analysis Kit (Biosharp, Cat# BL114A, China) following the manufacturer’s instructions. The stained cells were analyzed using a flow cytometer. Firstly, cell populations were gated based on their forward scatter (FSC) and side scatter (SSC) characteristics to exclude cell debris and ensure the selection of intact cells. Next, the cells were gated based on PI fluorescence signals (PI-A and PI-W) to analyze the DNA content. The proportion of cells in different phases of the cell cycle (G1, S, and G2/M) were analyzed using the Sync Wizard project in MODFIT software.

### Cell apoptosis analysis

The apoptosis of cells was analyzed using Annexin V-FITC Apoptosis Detection Kit (Biosharp, Cat# BL107A, China) following the manufacturer’s instructions. Briefly, IGF2BP3 knockdown or control U87 cells were seeded onto 6-cm plates at an appropriate cell density. Subsequently, cells were trypsinized, washed with PBS, and then resuspended in the binding buffer. Cells were then stained by Annexin V-FITC and PI. The stained cells were analyzed using a flow cytometer. The fluorescence emissions of Annexin V-FITC and PI were measured to determine the proportion of cells in different apoptotic stages.

### Flow cytometry analysis of BODIPY-C11 stain

IGF2BP3 knockdown or control U87 and HS683 cells were seeded into 6-well plates at a suitable density. Control cells were treated with or without 10 μM RSL3 (APExBIO, Cat# B6095, USA) for 24 h to induce oxidative stress. IGF2BP3 knockdown cells were treated with or without 100 μM deferoxamine (DFO) (MedChem Express, Cat# HY-B1625) for 24 h to inhibit ferroptosis. Following treatment, the cell culture media was aspirated, and cells were incubated with 1.5 μM BODIPY™ 581/591 C11 (Thermo Fisher, Cat# D3861, USA) for 30 minutes to label lipid peroxidation. After incubation, cells were washed with PBS to remove any unbound BODIPY-C11, then cells were trypsinized and resuspended in PBS. Afterwards, the cells were loaded onto a flow cytometer and excited using a 488 nm laser. The fluorescent intensity of the BODIPY-C11 signal was measured, and cell counts were recorded. Histograms were plotted based on the fluorescent intensity and cell counts to analyze lipid peroxidation levels in the different treatment groups.

### Reactive oxygen species assay

IGF2BP3 knockdown or control U87 and HS683 cells were seeded into 6-well plates at an appropriate density. To measure the ROS levels, a Reactive Oxygen Species Assay Kit (Biosharp, Cat# BL714A, China) was utilized following the manufacturer’s instructions. Control cells were treated with or without 100 μM Rosup for 20 min to induce ROS generation. After the treatment, cells were incubated with 10 μM DCFH-DA (2’,7’-dichlorofluorescin diacetate) in serum-free DMEM for 20 minutes. Subsequently, cells were washed three times with serum-free DMEM to remove any unbound DCFH-DA. Cells were observed by microscope directly or trypsinized and resuspended in PBS for subsequent flow cytometry analysis.

### Immunofluorescent

Cells were seeded onto 6-well plates containing coverslips. When the cell confluency reached approximately 80%, the cells were fixed with 4% paraformaldehyde. Subsequently, the cells were washed with PBS and permeabilized using 0.1% Triton X-100. After another wash with PBS, the cells were blocked with 1% BSA to prevent nonspecific binding. Next, the cells were incubated with the primary antibody at 4°C overnight. Following the overnight incubation, the cells were washed again with PBS to remove any unbound primary antibody. Subsequently, the cells were incubated with the secondary antibody labeled with Alexa Fluor 488 (Absin, Cat# abs20013, China) or 594 (Absin, Cat# abs20021, China) in the dark at room temperature for 1 hour. The coverslips were mounted using Antifade Mounting Medium with Hoechst 33342 (Beyotime, Cat# C1025, China). Confocal microscopy was performed to capture fluorescent images of the stained cells.

### Multi-color Immunohistochemistry (mIHC)

The glioma tissue microarray (TMA) chip containing 60 clinical glioma patients’ tumor samples from Sun Yat-Sen Memorial Hospital, Sun Yat-Sen University, was used for mIHC. The mIHC was performed using the Four-color multiple fluorescent immunohistochemical staining kit (Absin, Cat# abs50012, China) following the manufacturer’s instructions. Briefly, the TMA slide was immersed in a xylene bath to remove the paraffin, followed by sequential immersion in fresh 100%, 95%, and 70% ethanol to rehydrate the tissues. For antigen retrieval, the slide was placed in a microwave-resistant plastic staining jar containing antigen retrieval solution. After blocking with 5% BSA, the slide was incubated with anti-GPX4 (Cell Signaling Technology, Cat# 52455, USA) primary antibody at room temperature for 30 minutes followed by three TBST washes. Next, the slide was incubated with the HRP-labeled secondary antibody. After another round of three TBST washes, the slide was stained by the Tyramide Signal Amplification (TSA) fluorescent dye. The immunofluorescence staining procedure was then repeated for the anti-IGF2BP3 (Signalway Antibody, Cat# 54968, USA) and anti-NRF2 (Cell Signaling Technology, Cat# 12721, USA) primary antibodies incubation, along with their respective fluorescent dyes staining. Following the incubation with the three primary antibodies and fluorescent dyes, the slide was subjected to DAPI staining to label cell nuclei. The fluorescent images of the stained TMA slide were scanned using the Pannoramic MIDI II system. The fluorescent images were captured by SlideViewer software. The fluorescent intensity (IOD) was quantified by Image-Pro Plus.

### Western blot

Cells were lysed using Cell lysis buffer for Western and IP (Beyotime, Cat# P0013, China) to extract the cellular proteins. The protein concentration in the cell lysate was determined using the Bradford Protein Assay Kit (Beyotime, Cat# P0006C, China). Equal amounts of cell lysate protein were loaded onto a 12% SDS gel and transferred to PVDF membrane. The membrane was incubated with corresponding specific primary: anti-NRF2 (Cell Signaling Technology, Cat# 12721, USA), anti-β-Actin (Proteintech, Cat# 66009-1-Ig, China), anti-β-Tubulin (Cell Signaling Technology, Cat# 86298, USA), anti-IGF2BP3 (Signalway Antibody, Cat# 54968, USA), anti-GPX4 (Cell Signaling Technology, Cat# 52455, USA). Following the primary antibody incubation, the membrane was washed with TBST and incubated with the appropriate secondary antibodies: HRP Goat Anti Mouse IgG (Immunoway, Cat# RS0001), or HRP-labeled Goat Anti-Rabbit IgG (EpiZyme, Cat# LF102). MCE Ultra High Sensitivity ECL Kit (MedChem Express, Cat# HY-K1005) was used to detect the presence of HRP. The results were visualized using GelView 1500 ProII imaging system.

### RNA pull-down

The RNA sequences for RNA pull-down assay were:

m6A_143_: 5’_Biotin_UCCGCCAA**GG(m6A)CA**UCGAC_3’;

A_143_: 5’_Biotin_UCCGCCAA**GGACA**UCGAC_3’;

m6A_575_: 5’_Biotin_ AGAA**GG(m6A)CC**UGCCCCACUAUUU_3’;

A_575_: 5’_Biotin_ AGAA**GGACC**UGCCCCACUAUUU_3’;

m6A_694_: 5’_Biotin- GGC**AG(m6A)CC**CGAAAAUC_3’;

A_694_: 5’_Biotin-GGC**AGACC**CGAAAAUC_3’.

A total of 500 pmol of each RNA sequence was denatured by heating at 90 °C for 5 min, followed by immediate placement on ice. Streptavidin-coupled Dynabeads (Invitrogen, Cat# 65801D, USA) were precleared using binding buffer (20 mM Tris-HCl pH 7.5, 150 mM NaCl, 5 mM KCl, 5 mM MgCl_2_, 0.1% NP-40, 1 mM DTT, 4 U RNA inhibitor). Streptavidin magnetic beads and biotin labeled RNA were mixed by rotating at 4 °C for 2 h, then washed by binding buffer. Next, the streptavidin magnetic beads with bound RNA were mixed with U87 or HS683 cell lysate and incubated by rotating at 4 °C for 4 h. After washing by binding buffer, the beads were eluted by SDS-PAGE Sample Loading Buffer (Beyotime, Cat# P0015, China) and anti-IGF2BP3 western blot was performed.

### Methylated (m6A) RNA immunoprecipitation (MeRIP)

The MeRIP assay was conducted using the Magna MeRIP™ m6A Kit (Sigma, Cat# 17-10499, USA), following the manufacturer’s instructions. The mRNA was purified from total RNA using the mRNA Purification Kit (Beyotime, Cat# R0071M, China). In brief, 5 mg of mRNA was incubated with anti-m6A antibody-coupled magnetic beads, while mouse IgG-coupled beads were utilized as the negative control. The m6A-enriched mRNA was eluted using 20 mM *N*^*6*^-methyladenosine. RNA Clean Magnetic Beads (Beyotime, Cat# R0081, China) were employed to further purify the enriched mRNA. Real-time quantitative PCR was performed to assess the level of m6A enrichment in mRNA.

### RNA stability

HEK 293 T cells were subjected to lentiviral infection with either GPX4-WT or GPX4-A575T lentivirus to achieve the overexpression of the respective genes. Cells were treated with 10 μg/mL Actinomycin D (APExBIO, Cat# A4448, USA) and collected after 3 and 6 h. Total RNA was extracted, and real-time quantitative PCR (qPCR) was performed to quantify the levels of GPX4 mRNA at each time point. The baseline GPX4 mRNA level without Actinomycin D treatment was used as a reference, representing 100%. To assess RNA stability, the obtained qPCR data was used to plot a degradation line using linear regression approach.

### Cytoplasmic RNA extraction

Cytoplasmic RNA extraction was performed using the PARIS™ Kit (Invitrogen, Cat# AM1921, USA), following the manufacturer’s instructions. Briefly, the cells were lysed using Cell Fractionation Buffer. After lysis, the cellular components were separated by centrifugation, resulting in a pellet and a supernatant. The supernatant contained the cytoplasmic fraction. The cytoplasmic lysate was mixed with 2x Lysis/Binding Solution and 100% ethanol. The mixture was then loaded onto a Filter Cartridge, and cytoplasmic RNA was eluted by elution buffer.

### Xenograft model

The 6-weeks ages of male BALB/c Nude Mouse were used in this study. The animal experiment protocol had been reviewed and approved by the Animal Research Ethics Committee of the Affiliated Hospital of Guangdong Medial University (protocol:AHGDMU-LAC-I(1)-2208-A014). To establish the xenograft model, 5 ×10^6^ U87 control cells or U87 IGF2BP3-KD cells were subcutaneously injected into the rear flank of nude mice (four mice per group). Tumor growth was monitored every two days by measuring the tumor size using a caliper. Once the diameter of the tumor reached approximately 10 mm, the mice were sacrificed, and the tumors were excised and weighed. Tumor volume was calculated using the formula V = (W^2^ × L) / 2, where V represents the tumor volume, W indicates the tumor width, and L denotes the tumor length.

### Immunohistochemistry (IHC)

Tumors from xenograft models and normal mouse brains were fixed with 4% paraformaldehyde and dehydrated using a gradient concentration of ethanol. The tissues were then embedded in paraffin, sliced into thin sections, and mounted on slides. The slides were immersed in a xylene bath to remove the paraffin, followed by sequential immersion in a descending ethanol series to rehydrate the tissues. To retrieve antigens for better antibody binding, the slides were placed in a microwave-resistant plastic staining jar containing antigen retrieval solution. To prevent non-specific staining, the slides were treated with 3% hydrogen peroxide (H_2_O_2_) to inactivate endogenous peroxidase activity. The slides were incubated with anti-IGF2BP3 (Signalway Antibody, Cat# 54968, USA) antibody followed by three PBST washes. Next, the slides were incubated with the HRP-labeled secondary antibody. To visualize the presence of the target protein, diaminobenzidine (DAB) was applied to the slides, leading to the formation of a brown color at the site of antibody binding. Slides were washed by tap water and stained by hematoxylin. A light microscope was utilized to conduct histopathological assessment and analyze immunohistochemistry staining. The staining quantification was performed using Image-Pro Plus software.

### Human microglia cells and glioma cells co-culture

To examine the phagocytosis of glioma cells by human microglia (HM) cells, mCherry stably transduced HM cells were co-cultured with GFP stably transduced IGF2BP3-KD or control U87 and HS683 cells. An equal number of HM cells and glioma cells were mixed and seeded into 6-well plates. After three days of co-culture, interaction between HM cells and glioma cells was visualized and analyzed using confocal microscopy to investigate the phagocytic activity of HM cells towards glioma cells.

To investigate the influence of IGF2BP3 in glioma cells on human microglia cells, co-culture experiments were conducted using a transwell system. IGF2BP3-KD or control U87 and HS683 glioma cells were seeded into the upper chamber, while human microglia cells were seeded into the bottom chamber. After three days of co-culture, the human microglia cells from the bottom chamber were lysed, and protein extracts were subjected to western blot analysis.

### Statistical analysis

The results of real time quantitative PCR, colony formation, and immunofluorescent were analyzed using paired two-tailed t-test. The results of CCK-8, cell cycle, and cell apoptosis were analyzed using multiple t-tests. The correlation between GPX4 and IGF2BP3 mRNA expression were analyzed using Pearson correlation. The association between IGF2BP3 and GPX4 or NRF2 expression was determined using a linear regression approach. In all analyses, *p* < 0.05 were considered statistically significant.

### Supplementary information

Supplementary Figures_clean version

Supplementary tables

Original Data File

reproducibility checklist

## Data Availability

All primary data presented in this study are available upon reasonable request to the corresponding authors.
