# Supplementary material for: Depletion of the N6-Methyladenosine (m6A) reader protein IGF2BP3 induces ferroptosis in glioma by modulating the expression of GPX4
Source: Cell Death Dis. 2024 Mar 1;15(3):181. doi: 10.1038/s41419-024-06486-z (PMC10907351; doi:10.1038/s41419-024-06486-z)
Supplement: Supplementary file 1 — Supplementary Figures_clean version [file 41419_2024_6486_MOESM1_ESM.pdf]

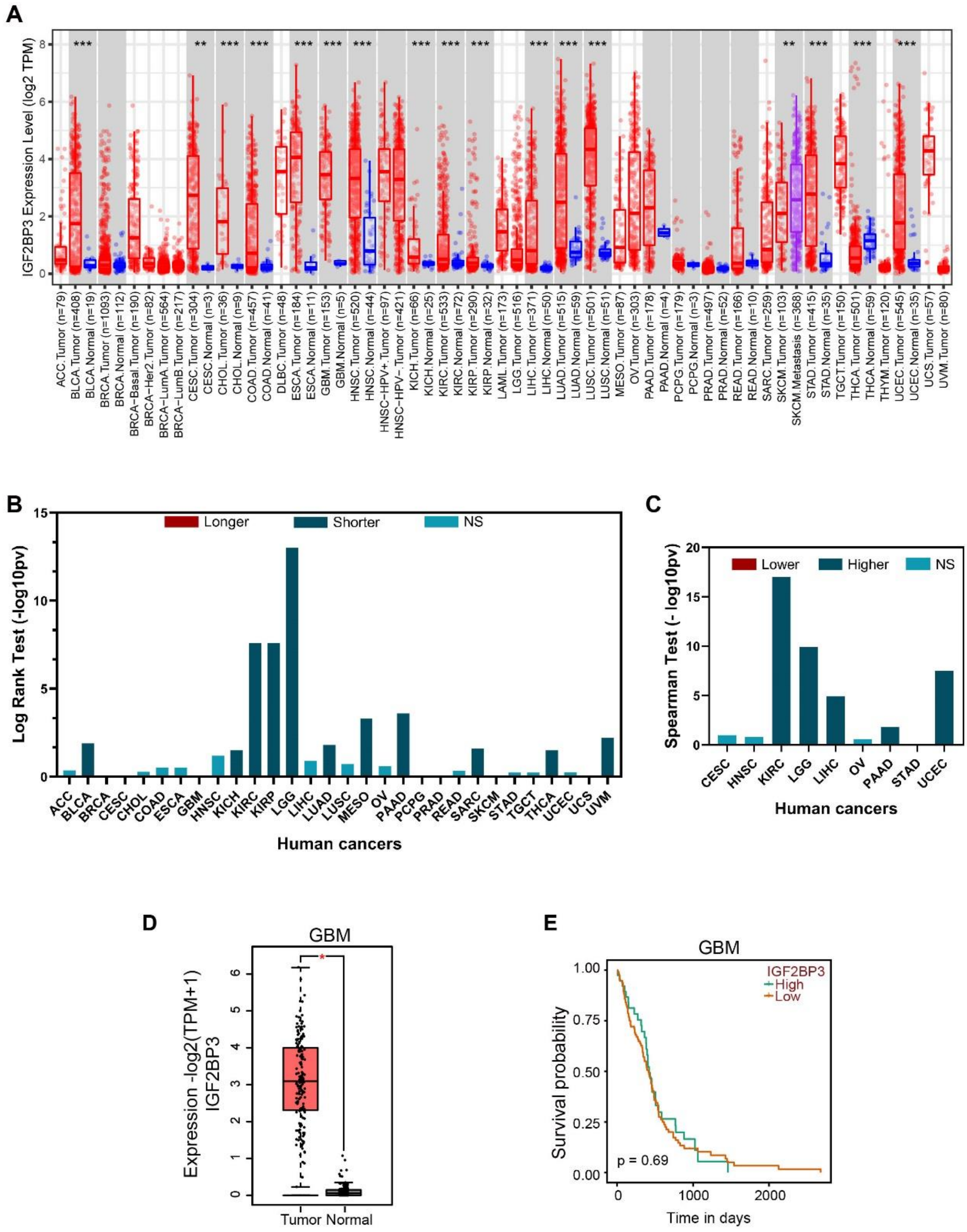

Supplementary Figure 1. Pan-cancer analysis of IGF2BP3 expression. A IGF2BP3 mRNA expression profile in tumor and normal tissues form TCGA database. **B** Correlation between IGF2BP3 expression and overall survival across human cancers. The red color indicates longer survival time, blue represents shorter survival time, and cyan denotes no significant difference. **C** Associations between IGF2BP3 expression and grade across human cancers. Red indicates gene expression is associated with lower grade, blue indicates association with higher grade, and cyan denotes no significant association. **D** Expression distribution of IGF2BP3 in tumor and normal tissues in GBM patients from TCGA database. **E** KM curve showing overall survival for GBM patients from TCGA database with high and low expression of IGF2BP3.

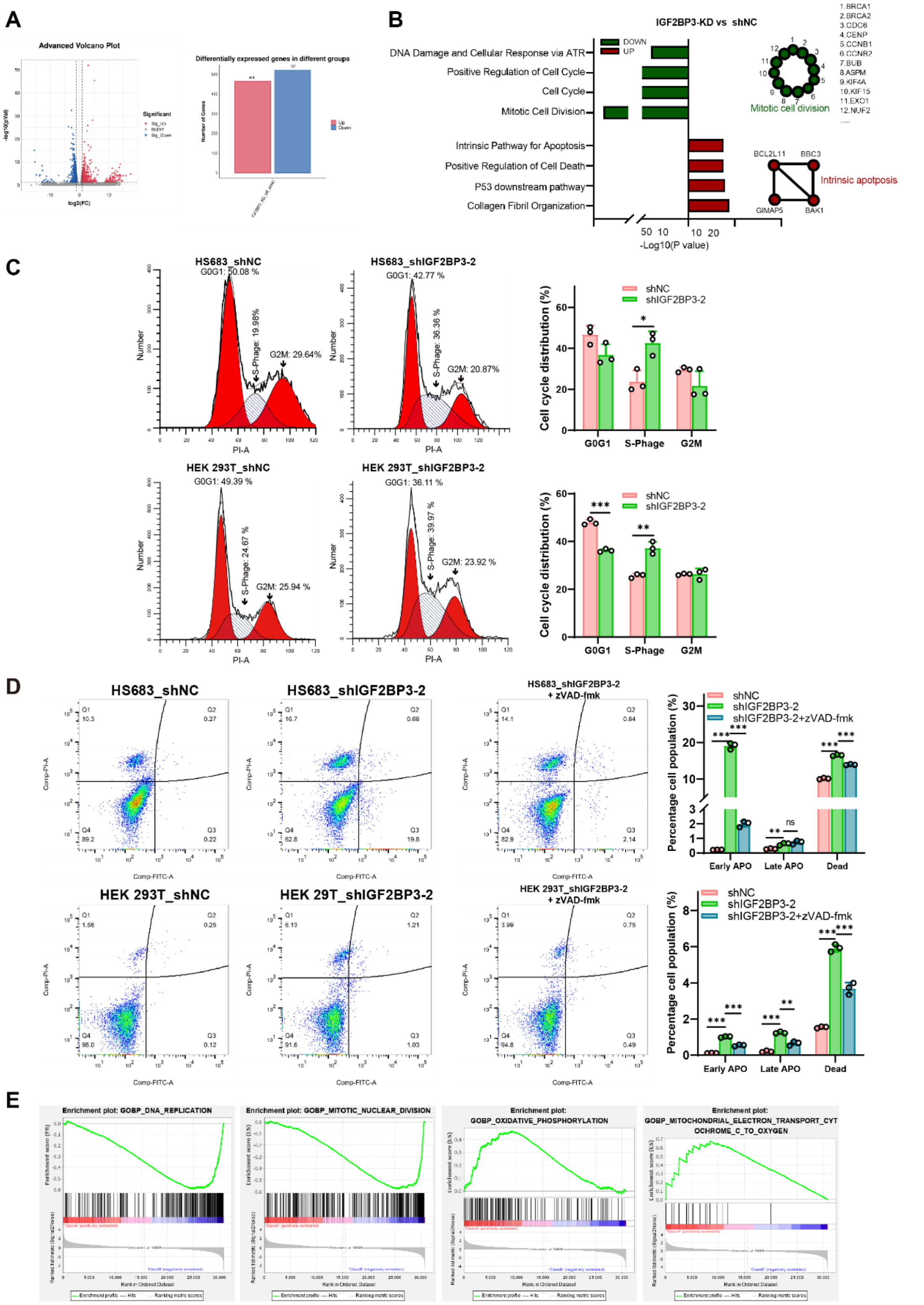

Supplementary Figure 2. Differential gene expression and pathway enrichment in U87 cells upon IGF2BP3 knockdown. A Volcano diagram and column graph displaying DEGs in U87 IGF2BP3-KD vs shNC cells. **B** GO enrichment analysis of DEGs. The top 4 enriched functional categories with the lowest p-values are displayed. **C** Cell cycle analysis showing IGF2BP3 knockdown led to S-phase arrest in HS683 and HEK 293T cells. Multiple t-test was used to compare the results (n = 3). **D** Cell

apoptosis analysis demonstrating an increase in cell apoptosis and cell death upon IGF2BP3 knockdown in HS683 and HEK 293T cells. Adding of apoptosis inhibitor zVAD-fmk resulted in partial mitigation of the apoptosis triggered by IGF2BP3 knockdown (Multiple t-test). **E** GSEA showing enriched signaling pathways upon IGF2BP3 knockdown in U87 cells.

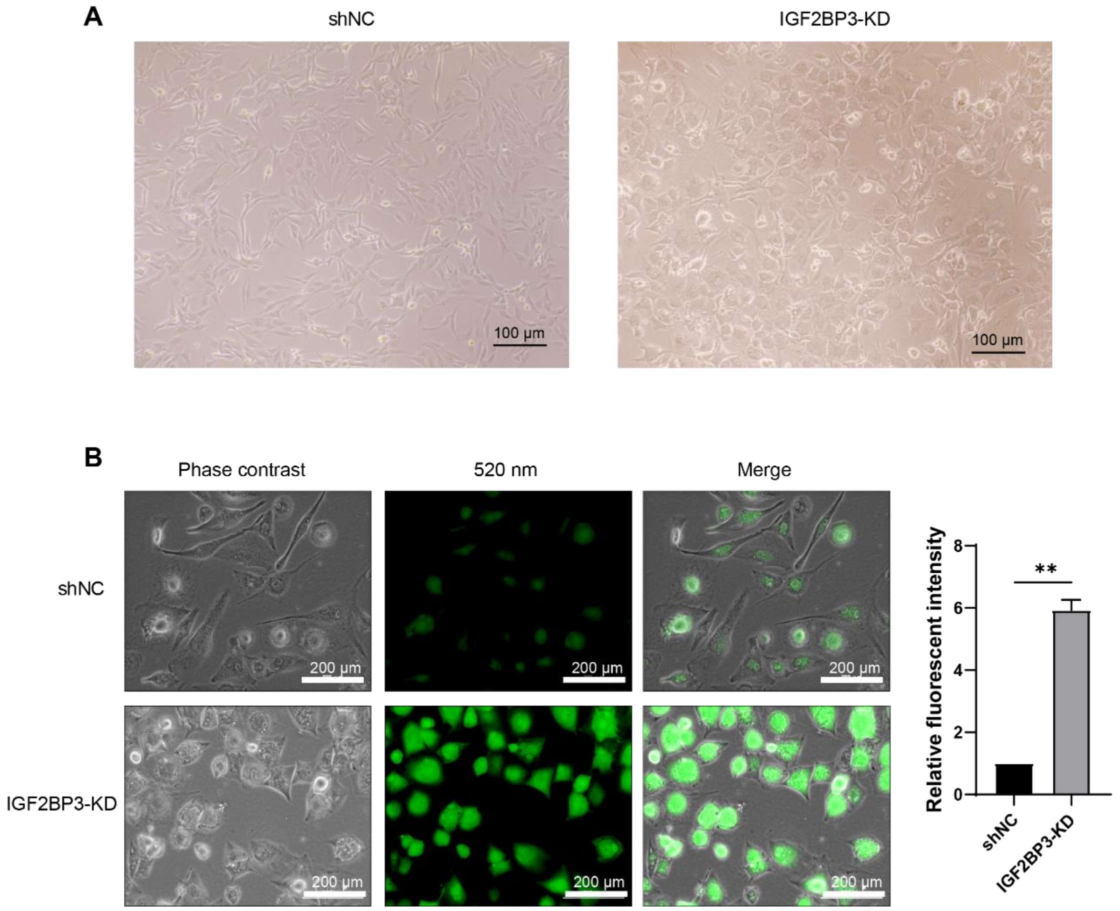

Supplementary Figure 3. Induction of ferroptosis phenotype upon knockdown of IGF2BP3 in U87 Cells. A U87 cells morphology changes upon IGF2BP3 knockdown. **B** Measurement of ROS levels in U87 IGF2BP3-KD cells and shNC cells using DCFH-DA probe. The fluorescent intensity is quantified using Image-Pro Plus software and analyzed by paired two-tailed t-test. Data are presented as the mean ± standard deviation (SD) from three independent experiments. ***$p < 0.001$, **$p < 0.01$, *$p < 0.05$.

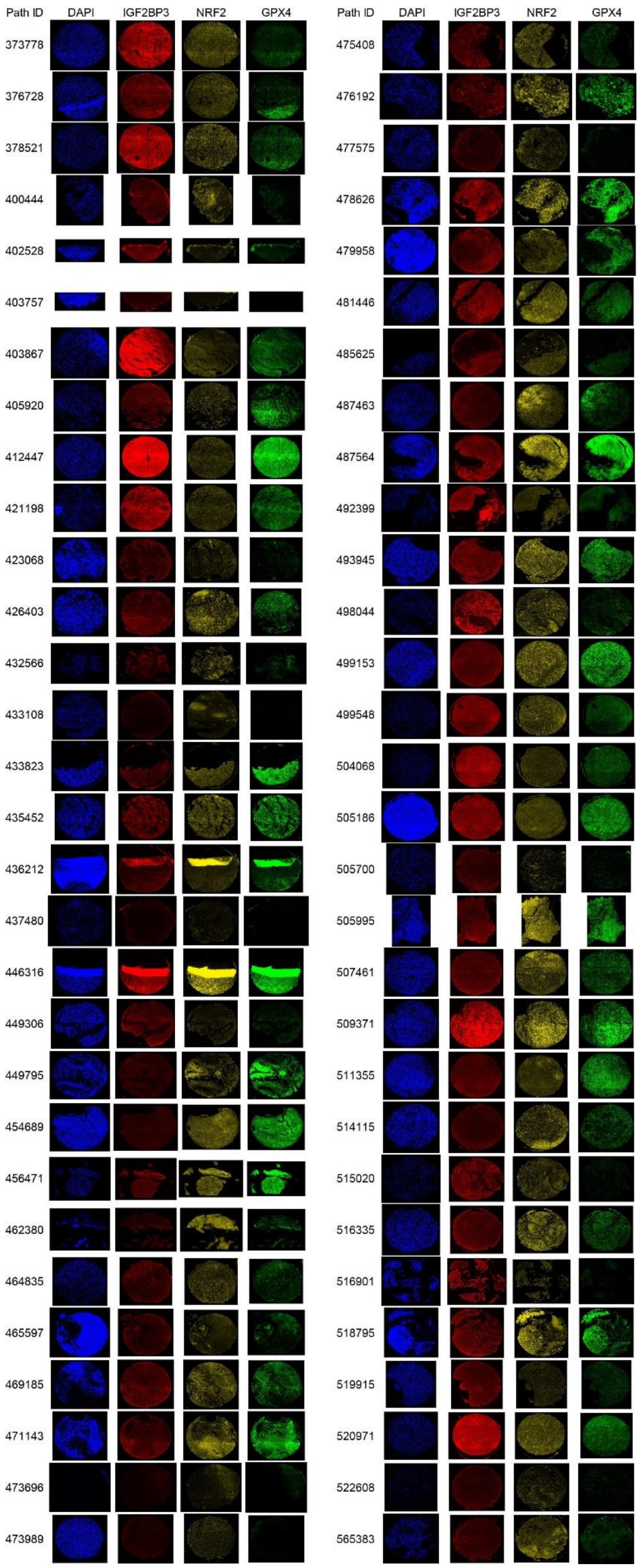
Path ID
DAPI
IGF2BP3
NRF2
GPX4
373778
376728
378521
400444
402528
403757
403867
405920
412447
421198
423068
426403
432566
433108
433823
435452
436212
437480
446316
449306
449795
454689
456471
462380
464835
465597
469185
471143
473696
473989
475408
476192
477575
478626
479958
481446
485625
487463
487564
492399
493945
498044
499153
499548
504068
505186
505700
505995
507461
509371
511355
514115
515020
516335
516901
518795
519915
520971
522608
565383

34

Supplementary Figure 4. mIHC analysis of IGF2BP3, NRF2, and GPX4 expression in a glioma TMA Chip. mIHC was performed to detect the expression levels of IGF2BP3, NRF2, and GPX4 in a glioma tissue microarray (TMA) chip containing 60 clinical glioma patients' tumor samples. The fluorescent intensity revealed the protein expression levels, and the quantification was conducted using Image-Pro Plus software.

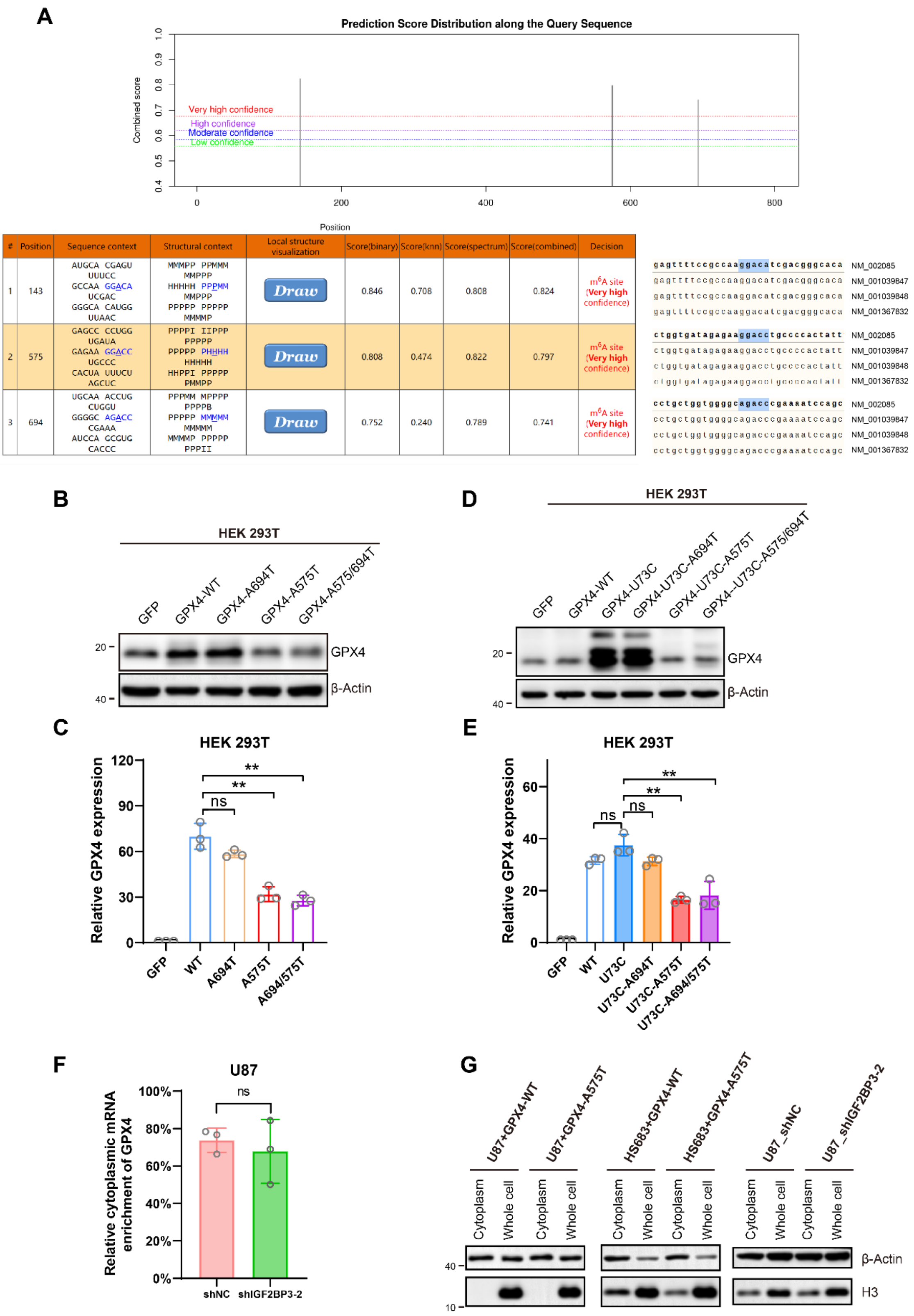

Supplementary Figure 5. Importance of nucleotide A575 in GPX4 transcript for GPX4 protein expression. **A** The online m6A modification site prediction tool (https://www.cuilab.cn/sramp) indicated three potential m6A modification sites in GPX4 CDS and 3'UTR regions. DNA alignment demonstrated that these three sites are conserved in all four GPX4 transcripts. **B-C** HEK 293T cells were infected with

lentivirus overexpressing GPX4-WT, GPX4-A575T, GPX4-A694T, or GPX4-A575/694T. The GPX4 protein and mRNA expression levels were verified using western blot (**B**) and qPCR (**C**), respectively. **D**-**E** HEK 293T cells were infected with lentivirus overexpressing GPX4-WT, GPX4-U73C, GPX4-U73C-A575T, GPX4-U73C-A694T, or GPX4-U73C-A575/694T. The GPX4 protein and mRNA expression levels were verified using western blot (**D**) and qPCR (**E**), respectively. **F** Cytoplasmic and whole cells' RNA of U87 IGF2BP3-KD cells and control cells were extracted, and the expression rate of GPX4 in the cytoplasm relative to the whole cells was verified using qPCR. **G** The cytoplasmic fraction separation efficiency was validated using western blot. β-Actin was used as a cytoplasmic marker, and H3 was used as a nuclear marker.

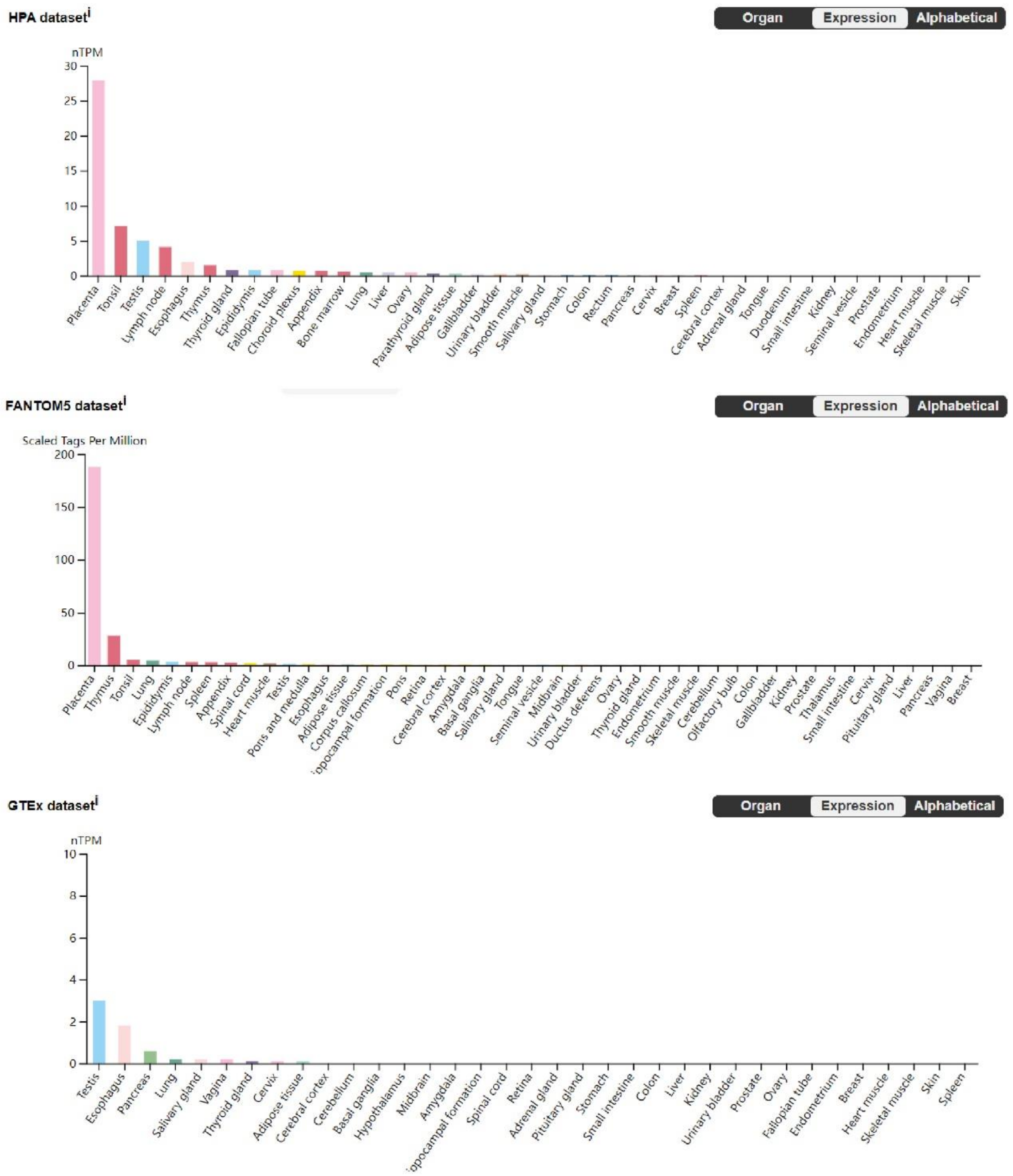

Supplementary Figure 6. Overview of IGF2BP3 mRNA expression in different human tissues from HPA, FANTOM5, and GTEx Dataset. The figure provides an overview of IGF2BP3 RNA expression levels in various human tissues, as obtained from three different datasets: the Human Protein Atlas (HPA) dataset, the FANTOM5 dataset, and the Genotype-Tissue Expression (GTEx) dataset.
