# Supplementary material for: Depletion of the N6-Methyladenosine (m6A) reader protein IGF2BP3 induces ferroptosis in glioma by modulating the expression of GPX4
Source: Cell Death Dis. 2024 Mar 1;15(3):181. doi: 10.1038/s41419-024-06486-z (PMC10907351; doi:10.1038/s41419-024-06486-z)
Supplement: Supplementary file 2 — Supplementary tables [file 41419_2024_6486_MOESM2_ESM.docx]

**Supplementary Table 1. List of the 22 m6A regulators that were tested for their prognostic value in human TCGA-LGG patients.**

| Category | Regulators involved | Abbreviations |
| --- | --- | --- |
| Writers | methyltransferase like 3  methyltransferase like 14  WT1-associated protein  methyltransferase 16  vir like m^6^A methyltransferase associated  zinc finger CCCH-Type containing 13  zinc finger CCHC domain containing 4  Casitas B-lineage lymphoma-transforming sequence‐like protein 1 | METTL3  METTL14  WTAP  METTL16  VIRMA  ZC3H13  ZCCHC4  CBLL1 |
| Erasers | α-ketoglutarate-dependent dioxygenase alkB homolog 5  fat mass- and obesity-associated protein | ALKBH5  FTO |
| Readers | putative RNA-binding protein 15  RNA binding motif protein 15B  insulin like growth factor 2 MRNA binding protein 1  insulin like growth factor 2 MRNA binding protein 2  insulin like growth factor 2 MRNA binding protein 3  YTH domain-containing 1  YTH domain-containing 2  YTHN6-methyl-adenosine RNA binding protein 1  YTHN6-methyl-adenosine RNA binding protein 2  YTHN6-methyl-adenosine RNA binding protein 3  heterogeneous nuclear ribonucleoprotein A2/B1  heterogeneous nuclear ribonucleoprotein C | RBM15  RBM15B  IGF2BP1  IGF2BP2  IGF2BP3  YTHDC1  YTHDC2  YTHDF1  YTHDF2  YTHDF3  HNRNPA2B1  HNRNPC |

**Supplementary Table 2. Statistical analysis of eight m6A regulators that were found to correlate with poor prognosis in human TCGA-LGG.**

| Gene | Cohort | Median survival time (months) | Hazard ratio (HR) | *p*-value |
| --- | --- | --- | --- | --- |
| ZCCHC4 | Low | 106.7 | 1.59 | 0.011 |
|  | High | 66.7 |  |  |
| RBM15 | Low | 119.0 | 2.00 | <0.0001 |
|  | High | 63.8 |  |  |
| IGF2BP2 | Low | 115.7 | 2.94 | <0.0001 |
|  | High | 63.0 |  |  |
| IGF2BP3 | Low | 135.6 | 3.40 | <0.0001 |
|  | High | 51.6 |  |  |
| YTHDC2 | Low | 106.7 | 1.92 | <0.0001 |
|  | High | 63.8 |  |  |
| YTHDF2 | Low | 106.7 | 2.23 | <0.0001 |
|  | High | 58.7 |  |  |
| HNRNPA2B1 | Low | 96.9 | 2.04 | <0.0001 |
|  | High | 52.8 |  |  |
| ALKBH5 | Low | 95.8 | 1.73 | 0.003 |
|  | High | 62.9 |  |  |

**Supplementary Table 3. Clinical features of TCGA-LGG in cohorts stratified by low or high IGF2BP3 expression.**

| Characteristic | Low expression of IGF2BP3 | High expression of IGF2BP3 | *p* value |
| --- | --- | --- | --- |
| n | 264 | 264 |  |
| WHO grade, n (%) |  |  | < 0.001 |
| G2 | 144 (30.8%) | 80 (17.1%) |  |
| G3 | 90 (19.3%) | 153 (32.8%) |  |
| IDH status, n (%) |  |  | < 0.001 |
| WT | 20 (3.8%) | 77 (14.7%) |  |
| Mut | 242 (46.1%) | 186 (35.4%) |  |
| 1p/19q codeletion, n (%) |  |  | < 0.001 |
| codel | 122 (23.1%) | 49 (9.3%) |  |
| non-codel | 142 (26.9%) | 215 (40.7%) |  |
