# Supplementary material for: Depletion of the N6-Methyladenosine (m6A) reader protein IGF2BP3 induces ferroptosis in glioma by modulating the expression of GPX4
Source: Cell Death Dis. 2024 Mar 1;15(3):181. doi: 10.1038/s41419-024-06486-z (PMC10907351; doi:10.1038/s41419-024-06486-z)

Figure 4A

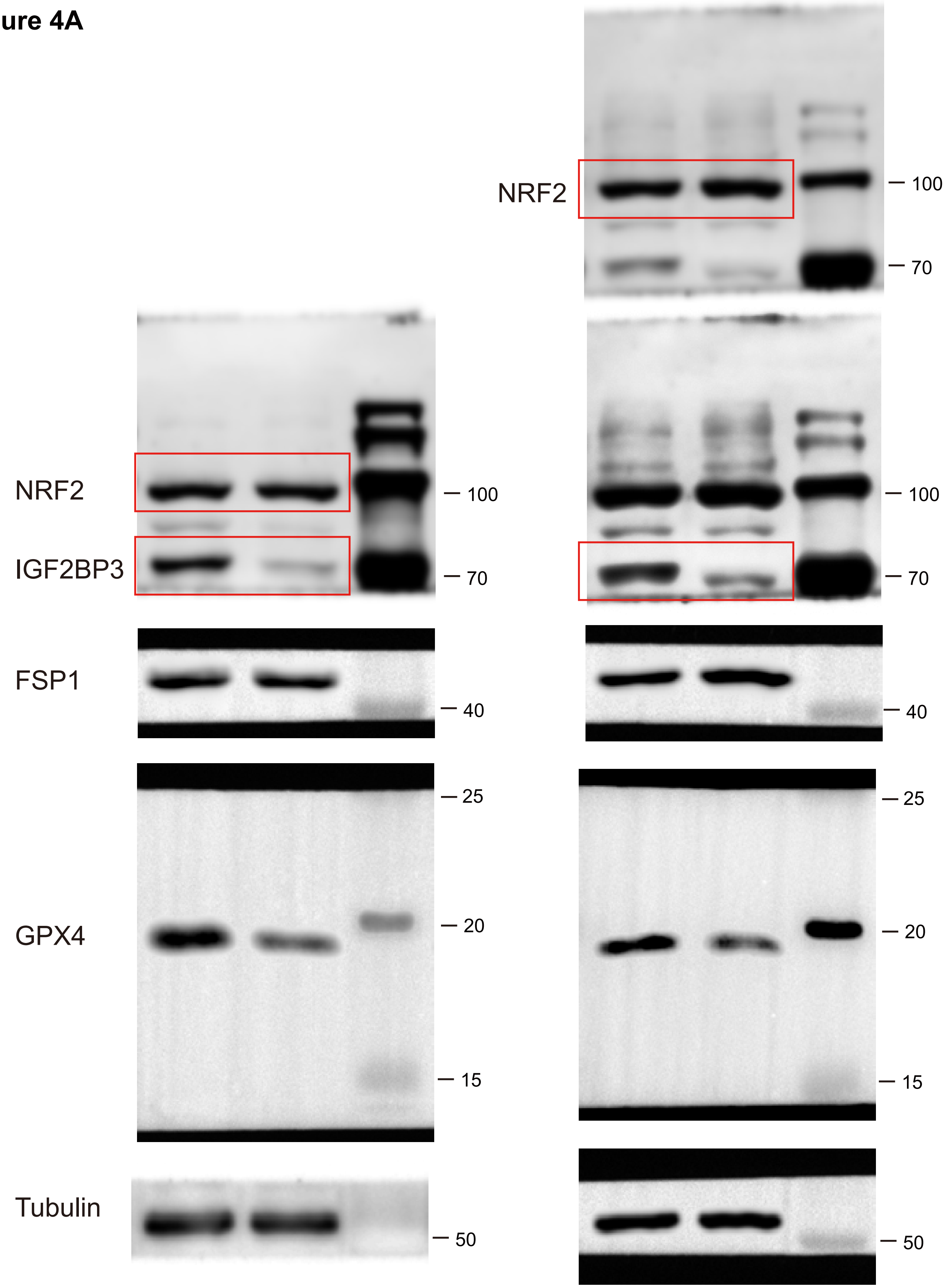

NRF2
100
70
NRF2
IGF2BP3
100
70
FSP1
40
40
GPX4
25
20
15
25
20
15
Tubulin
50
50

Figure 4C

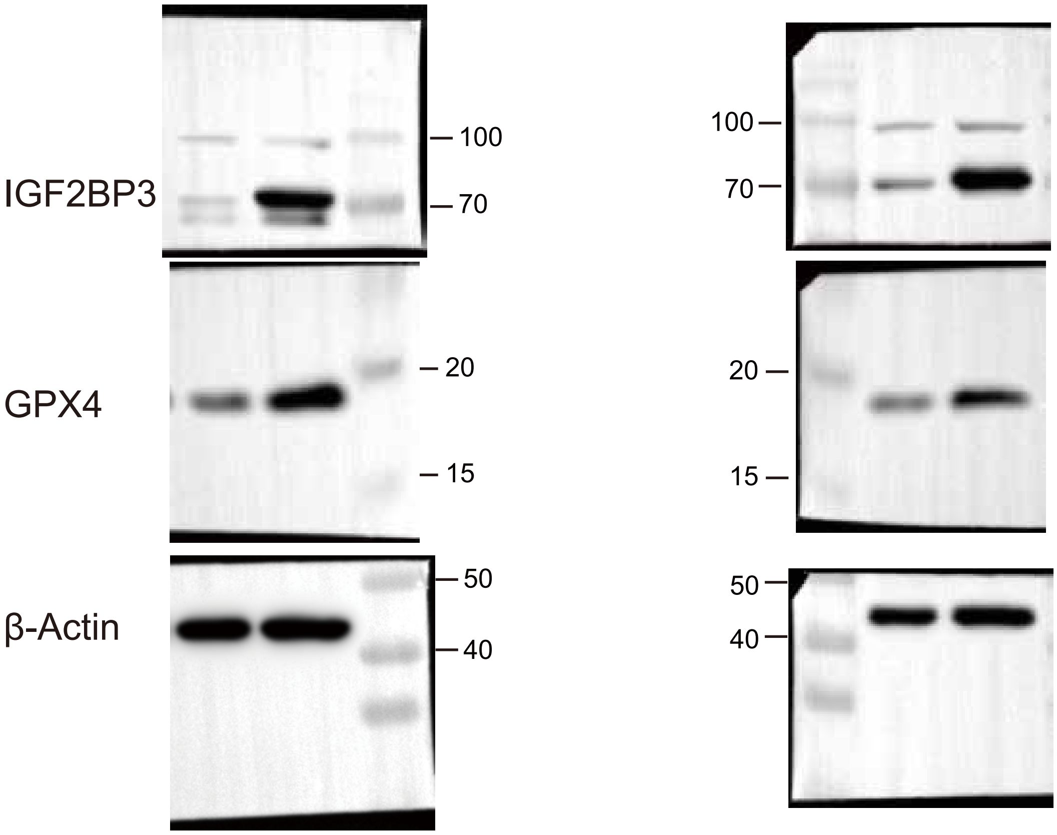

Figure 4E

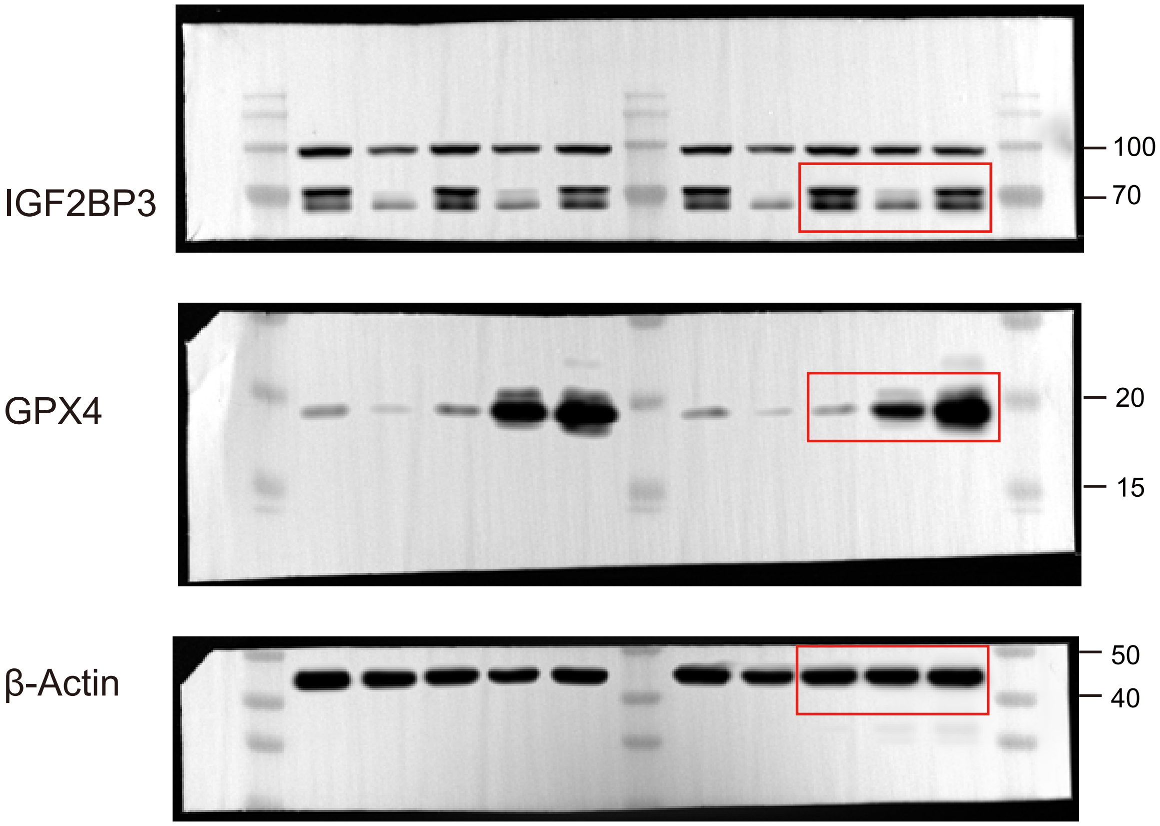

Figure 4F

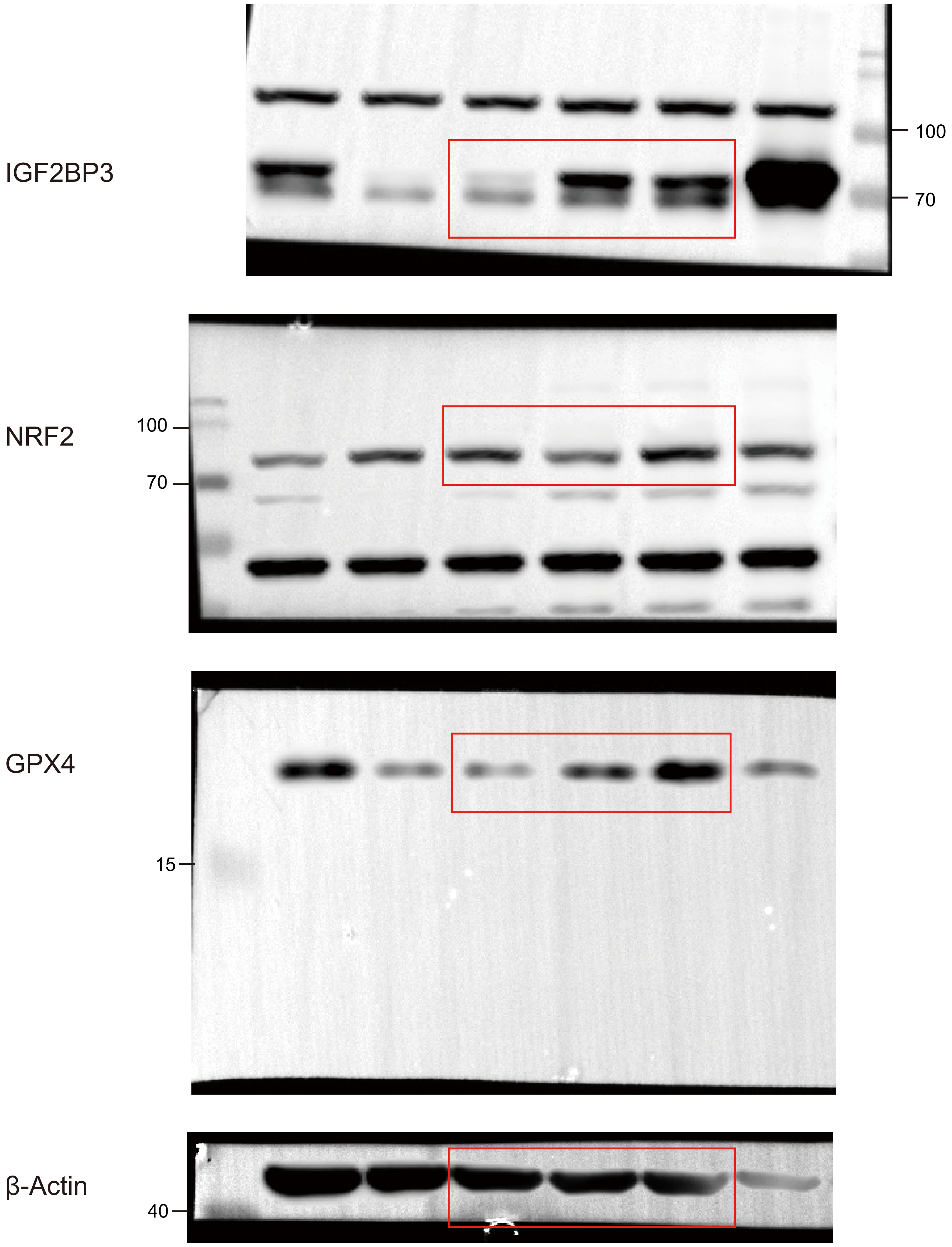

Figure 5B

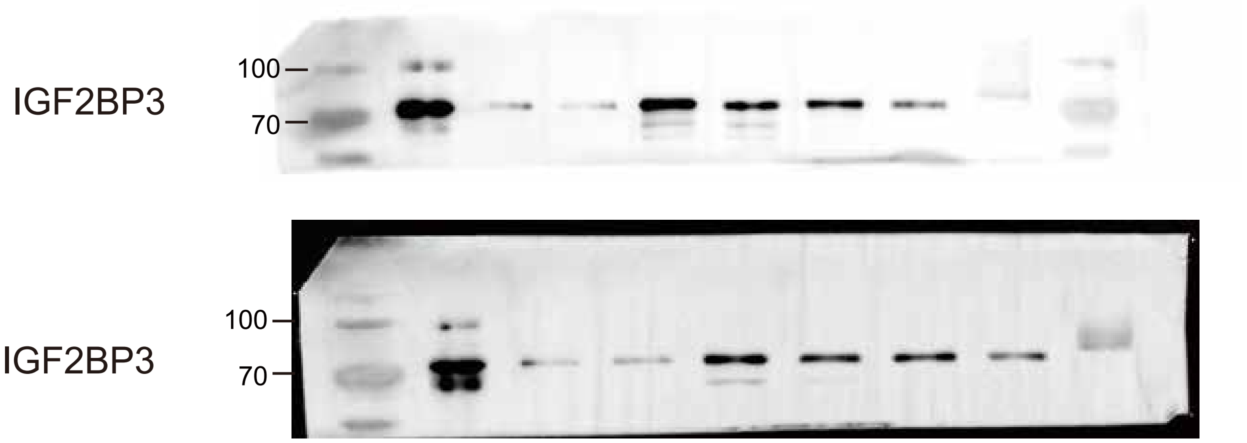

Figure 5D

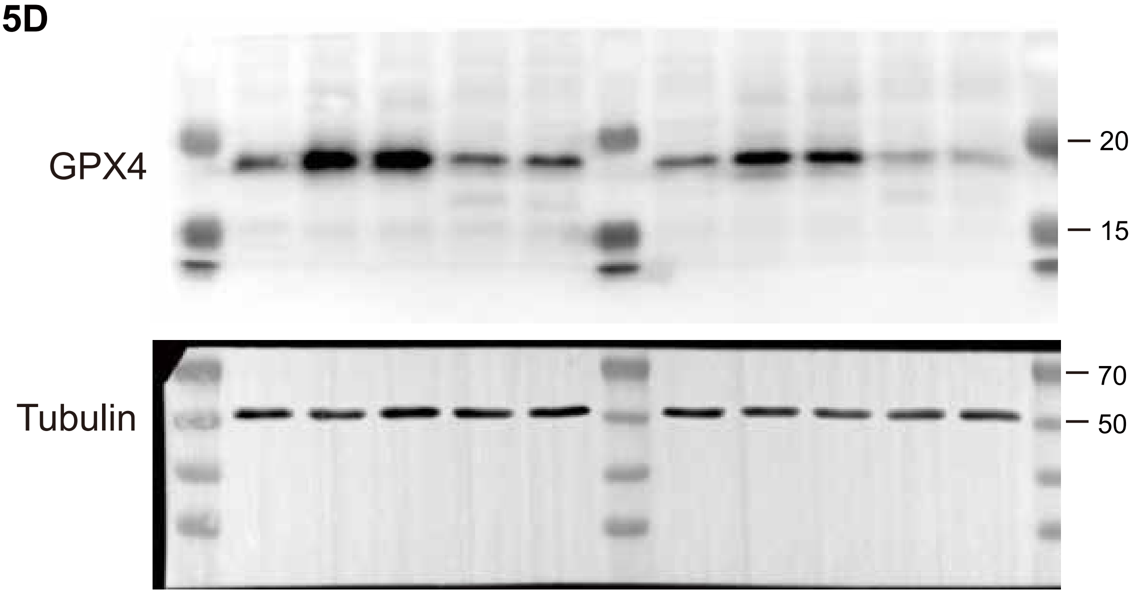

Figure 5F

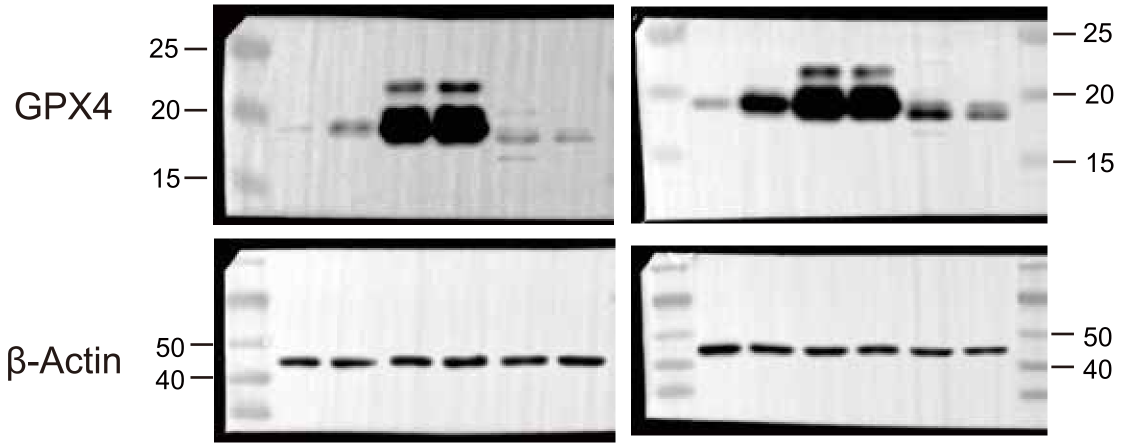

Figure 5J

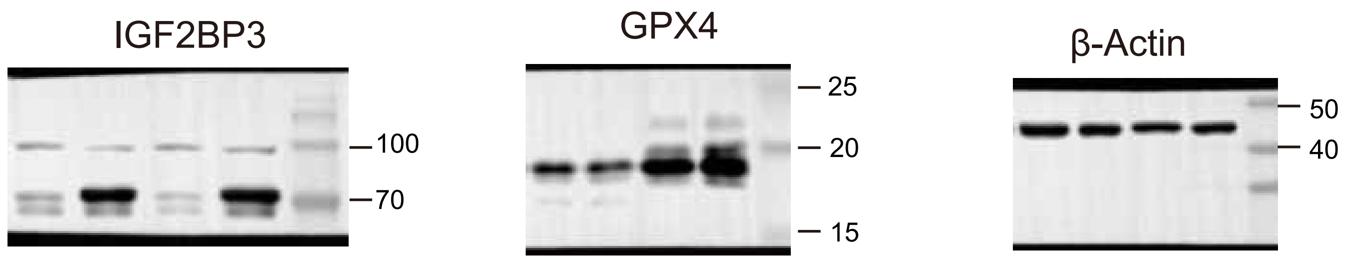

Figure 6E

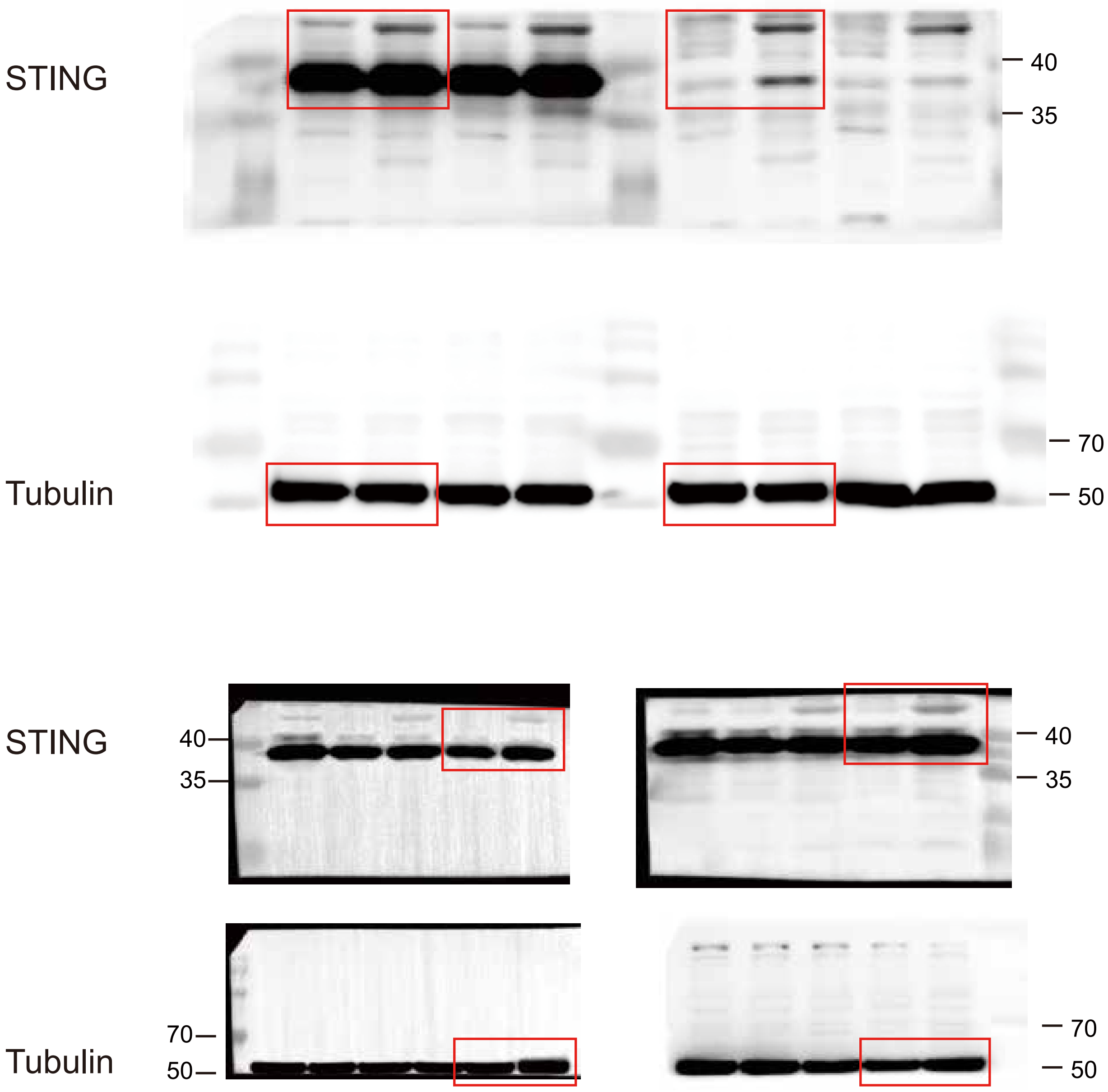

Supplementary Figure 5B

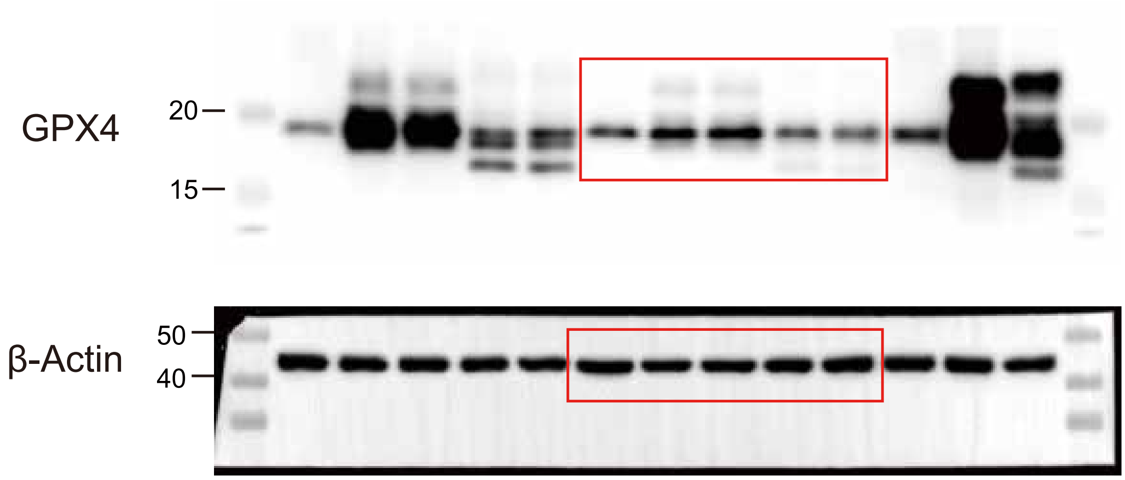

Supplementary Figure 5D

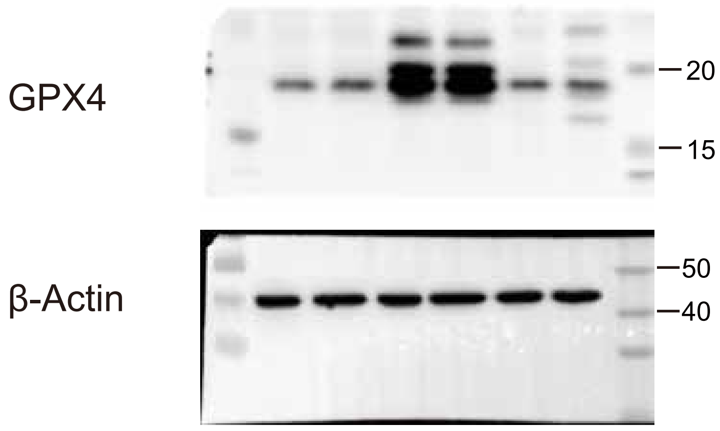

Supplementary Figure 5G

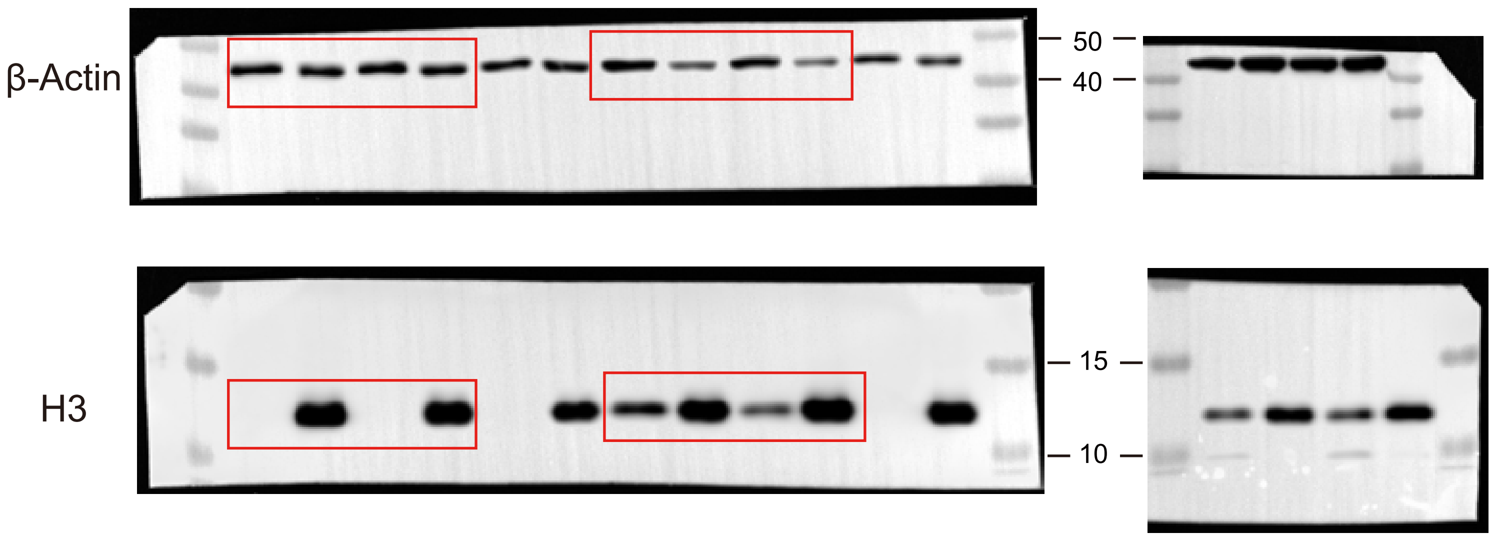

Supplement: Supplementary file 3 — Original Data File [file 41419_2024_6486_MOESM3_ESM.pdf]
